# Effect of a nutrient-rich, food-based supplement given to rural Vietnamese mothers prior to and/or during pregnancy on birth outcomes: A randomized controlled trial

**DOI:** 10.1371/journal.pone.0232197

**Published:** 2020-05-29

**Authors:** Hoang T. Nga, Phi N. Quyen, Benjamin W. Chaffee, Nguyen T. Diep Anh, Tu Ngu, Janet C. King

**Affiliations:** 1 National Institute of Nutrition, Hanoi, Vietnam; 2 University of California San Francisco, San Francisco, CA, United States of America; 3 Children’s Hospital Oakland Research Institute, Oakland, CA, United States of America; Institut de recherche pour le developpement, FRANCE

## Abstract

Obtaining a nutrient-rich diet during pregnancy is a challenge for pregnant women living in low-income countries. This randomized, controlled trial was designed to determine if a freshly prepared food supplement from local animal-source foods and dark-green leafy vegetables given prior to and/or during pregnancy improved birth outcomes in rural Vietnamese women. Primiparous women, 18 to 30 years of age, who participated in the study were assigned to one of three groups: PC-T women received the supplement from pre-conception to term, MG-T women received the supplement from mid-gestation to term, and the RPC women received routine prenatal care. Supplement intake was observed and quantified. Infant anthropometry was measured at birth and/or within seven days of delivery. The effect of the intervention on maternal and birth outcomes was determined using linear regression modeling. Of the 460 women enrolled in the study, 317 women completed the study. Those not completing the study had either moved from the area, did not conceive within 12 months of study enrollment, or miscarried. The food-based supplement increased protein, iron, zinc, folate, vitamin A and B12 intakes in the PC-T and the MG-T groups. However, it failed to alter infant anthropometric measurements at birth. In the entire cohort, maternal gestational weight gain was greater in women with a low pre-pregnancy BMI (<18.5) and in women with a higher educational attainment. Working as a farmer reduced gestational weight gain but it did not affect birth weight or length. In summary, a nutrient-rich, food-based supplement given to rural Vietnamese women from pre-conception to term or mid-gestation to term did not affect maternal or infant outcomes. The low weight gains, possibly due to demanding farm work done throughout the reproductive cycle, may have obviated any effects of the low energy, nutrient-rich food supplement on birth outcomes.

**Trial registration** : Registered Clinical Trials.gov: NCT01235767.

## Introduction

Infants may have a low birth weight because they were born too soon (premature) or were growth restricted *in utero*. Poor maternal nutrition is linked to both prematurity and fetal growth retardation. Indicators of maternal malnutrition before pregnancy, such as short stature, account for about one third of low weight births [1]. Attempts to improve birth weight through maternal supplementation with multiple micronutrients have had a modest effect [2, 3]. That may be due, in part, to the fact that the interventions were generally not initiated until after conception. Previous research shows that the nutritional status of the mother at conception is a key factor affecting fetal growth and pre-term deliveries [4–6]. This observation is verified by animal studies reporting that maternal nutrient deficiencies during the first weeks of pregnancy are linked to poor fetal growth [7]. Also, maternal diet at conception influences placental development and subsequent fetal growth [8].

Vietnam is in transition from a low to middle income country. During the past decade, the availability of prenatal health care improved as a result of national universal health coverage [9, 10]. Yet, the average birth weight remains unchanged, especially in rural populations [11]. In the late 1980s, country leadership initiated an integrated agricultural program to improve the diversity and quality of food crops produced and, therefore, the health and nutrition of the population [12, 13]. The program, called the VAC system (V for *Vuon* or garden, A for *Ao* or pond, and C for *Chuong* or cattle shed) supports the production of nutrient-rich foods by rural farmers. After nearly 30 years, the VAC program continues to be promoted as a cost-effective method for improving the nutrient intakes of rural women of reproductive age and their children. Although rural Vietnamese women often work as farmers growing VAC foods, their consumption of those foods is often limited because the foods are sold to urban dwellers for income [14]. Consequently, rural Vietnamese women of reproductive age report consuming diets low in the nutrients provided by VAC foods, i.e., iron, zinc, folate, vitamin B12, and vitamin A [15, 16].

Given the limited intake of micronutrient-rich foods and the demanding physical work performed by rural Vietnamese women [16, 17], we initiated a study to evaluate the impact of consuming a micronutrient-rich food supplement using the VAC foods on birth outcomes. Prenatal vitamin/mineral supplements are not routinely provided in rural Vietnam. Thus, a nutrient-rich food supplement provided by VAC foods could improve pregnancy outcomes. We hypothesized that the women receiving the VAC supplement from prior to conception to term would have better outcomes than that of the mothers receiving the VAC supplement only during the last half of pregnancy or given nutrition counseling instead of the supplement. As a secondary investigation, we hypothesized that the birth outcomes in the entire cohort is related to the mother’s baseline anthropometric measurements and her occupation as a farmer. Thus, the primary outcomes were birth weight, length, and prematurity. The effect of the VAC supplement on maternal weight gain and nutritional status was also measured.

## Methods

### Study setting and design

The VINAVAC study was done between 2011–2015 in Vietnam; VINA stands for Vietnam and VAC stands for the VAC system. Rural women were recruited for the study from 29 communes in the Cam Khe district, about 100 km northwest of Hanoi.

The protocol for this unmasked, randomized trial has been described elsewhere [18]. Briefly, non-pregnant women, 18–30 years of age, were recruited for the study when they registered to marry. Leaders of the women’s union in each commune identified women who were planning to marry and to have a child within one year. Those women were invited to come to the communal health station for screening. Women were excluded if they tested positive for pregnancy, if they had a history of illness such as HIV, tuberculosis, malaria, diabetes, heart or kidney diseases, if they had delivered a previous child, if they had a BMI <17, or if they were unable to commit to eating the food supplement every weekday morning at a specified site near their home.

After the consent was signed and the baseline measurements were completed, the women were randomly assigned to one of the three groups, PC-T: supplementation 5 days a week from study enrollment at pre-conception to term (~ 11 months total); MG-T: supplementation 5 days a week from mid-gestation to term (~ 5 months total); or RPC: routine prenatal care with no supplementation. A random number was generated for each participant using the random number generator in Excel (Microsoft, Redmond, WA, USA), and the participants from each commune were sorted by random numbers to assure each commune had an equal number of women in each of the three groups.

Women were discontinued from the study if they did not conceive after 1 year. Dietary, anthropometric, and blood analyses were measured at baseline/enrollment, mid, and late gestation (16±4 and 32±4 weeks, respectively).

### Ethics approval and consent to participate

The study protocol and consent forms were reviewed and approved by Children’s Hospital Oakland Institutional Review Board and by the National Institute of Nutrition in Hanoi, Vietnam. Written, informed consent was obtained from all of the participants.

### Preparation and delivery of the nutrient-rich, food-based supplement

Locally grown, dark-green leafy vegetables and animal source foods (i.e., pork meat, pork liver, pork blood, freshwater shrimp, or embryonated duck eggs) were included in the food supplement. A 10-day rotating cycle of recipes was developed to provide variety over the feeding period. The supplement provided at least 50% of a pregnant woman’s recommended dietary allowance for five nutrients: iron, zinc, folate, vitamin A, and vitamin B_12_ **(Table 1)**. The energy content of the supplement was limited to 190 kcal, or 10% of the daily energy requirement for Vietnamese women of reproductive age, to reduce displacement of other foods in the woman’s diet by the supplement. Composites of the 10 supplement menus were prepared for nutrient analysis. Iron and zinc levels were determined at Children’s Hospital Oakland Research Institute; vitamin A by Craft Technologies, Raleigh NC; and vitamin B_12_ and folate by Covance Inc., Battle Creek, MI. The average nutrient composition of 10-day menu cycle is shown in **Table 1**. The food supplement was prepared daily, Monday through Friday, according to specific protocols in three kitchens spaced throughout the Cam Khe District. The cooks purchased fresh ingredients at the local market and prepared the food items according to detailed, specific protocols. Individual portions were weighed into containers. At about 9 a.m., Healthcare Workers transported the food to designated eating sites for groups of the participants, observed food consumption, weighed any uneaten portion, and recorded the amount not consumed and why in a logbook. If a participant could not come to the eating site, the Health Worker brought the supplement to the participant’s home or her workplace and monitored and recorded intake as usual. Study team members observed aspects of the food preparation and consumption procedures on a random basis. The kitchen scales (2 per kitchen) and the 29 food scales for weighing uneaten food were checked weekly with a standard weight.

**Table 1:** Nutrient composition of the 10-day rotating menus composites^1^.

	Mean	Stand. Dev.	% of the RDA^2^
Portion size, cooked, g	257.4	14.3	N/A
Energy, kcal	193.4	52.4	10.4^3^
Protein, g	23.8	5.0	51.7
Vitamin A, μg	1,542	1,308	220.3
Folate, μg	407.0	73.7	101.7
Vitamin B_12_, μg	7.6	8.0	316.7
Iron, mg	15.5	10.2	86.1
Zinc, mg	5.2	3.1	65.0

^1^ Nutrient composition was analyzed: Energy by method of FAO 2003 [19]; Protein by AOAC 991.20 [20]; Iron and zinc levels by inductively-coupled plasma; vitamin A by Craft Technologies (http://www.crafttechnologies.com), and vitamins B_12_ and folate by Covance, Inc. [21, 22]

^2^ Recommended Dietary Allowance for women 19 to 30 years of age [23].

^3^ Percentage of Estimated Energy Requirement (EER) for women. EER = 354 - (6.91 × age[y]) + PA × [(9.36 × weight [kg]) + (726 × height[m])], where the age, weight and height of women were 21.5 years, 46.1 kg and 1.526 m, respectively and PA (physical activity coefficient) was 1.27 [24].

### Maternal measurements

Dietary intakes were assessed from two 24-h recalls done on non-consecutive days at three time-points in the study: at enrollment, at mid-pregnancy (16 weeks), and at late-pregnancy (32 weeks). At baseline, recalls were completed 4 weeks apart. During pregnancy, the diet recalls were done within a 6-week window at 16 and 32 weeks, i.e., ±3 weeks. In total, 2 recalls were completed per participant at 95% of the time-point visits. Trained staff did the recalls at the participants’ home. Food scales, a set of commonly used utensils (e.g., spoons, bowls, and cups), food pictures, and common mixed food recipes were used to estimate the amount of food consumed. The dietary recalls were coded using Vietnamese food composition tables [25], and the daily intakes of energy, macronutrients, vitamins and minerals were calculated. We did not exclude women who chose to take multiple micronutrients or iron/folate supplements during pregnancy.

At baseline, mid and late pregnancy, study staff completed anthropometric measurements and collected a fasting blood sample at the communal health station. The anthropometric measurements, done in duplicate, included the woman’s height, weight, mid-upper arm circumference, triceps and subscapular skinfold thickness [26]. The mid-upper-arm fat area (AFA) was calculated from measurements of mid-upper-arm circumference (MUAC) (mm) and triceps skinfold thickness (TSK) (mm): (TSK x MUAC/2)–(π x TSK^2^/4). Mid-upper-arm muscle area (AMA) was calculated as AMA = [(MUAC-∏ x TSK)^2^/4∏] -6.5 [26].

The collected blood was processed immediately in the field laboratory. EDTA-preserved whole blood was collected to measure hemoglobin by the cyanmethemoglobin method using a Drew D3 auto analyzer (DREW Scientific, Dallas, Texas, United States). Anemia was defined as a hemoglobin concentration below 12 g/dL for nonpregnant women and 11 g/dL for pregnant women [27]. Twenty-three women with a hemoglobin level below 11 g/dL at enrollment were given a daily supplement of 60 mg of iron for two months as recommended by the WHO [28] and, they were kept in the study. Hemoglobin concentrations were measured after treatment for two months, and the levels were above 11 g/dL in all but one woman who received supplemental iron for two more months to correct her hemoglobin concentration. The remaining whole blood was centrifuged and, the plasma and serum samples were stored at -20°C. All samples were transferred from the field laboratory to Hanoi within 2 weeks and then stored at -80°C. Iron and zinc were analyzed by inductively coupled plasma optical emission spectrometry (ICP-OES) at Children’s Hospital Oakland Research Institute [29]. Serum folate and vitamin B_12_ (cobalamin) were measured by microbiological assay using a chloramphenicol resistant strain of *Lactobacillus casei* and a colistin sulphate resistant strain of *Lactobacillus leichmannii* for folate and B_12_, respectively [21, 22]. Serum folate concentrations below 10 nmol/L and serum cobalamin concentrations below 150 pmol/L were considered deficient [30]. Serum vitamin A was measured by liquid chromatography-mass spectrometry (LC-MS) method. Two acute phase proteins were measured: plasma C-reactive protein (CRP) and serum α-1-acid glycoprotein (AGP), using ELISA kits (Immunology Consultants Laboratories, Portland, OR). A CRP level >5 mg/L or an AGP levels >1 g/L were defined as an inflammatory state [31].

The head of the communal health station contacted all women twice a month for a morbidity interview and to check the menstruation data. Urinary pregnancy tests were done monthly using the dipstick analysis. Gestational age, in weeks, was calculated from the first day of the last menstruation period (LMP date) and the date of birth (DOB): (DOB–LMP date)/7.

### Infant measurements at birth

The hospital staff in the delivery room measured infant birth weight immediately after the baby was cleaned. The baby was wrapped in a towel when weighed and then the towel weight was subtracted from the total. Study staff used standard weights to check the scale before using. The study staff measured the infant weight, length, head and mid-upper arm circumference in 199 infants (63% of the cohort) within seven days of delivery using standard WHO procedures [32]. Infants were weighed to the nearest 5 g without clothing using a digital baby scale (Seca 334, Seca Corporation, Hanover, MD). Infant length was measured using a portable infantometer (Seca 417), and head and mid upper-arm circumferences were measured with a Seca 212 tape. All measurements were recorded to the nearest 1 mm. Low birth weight was defined as < 2,500 g. Maternal gestational weight gain (GWG) was calculated as the difference between the weight at enrollment prior to conception and the weight at 32 weeks gestation.

### Statistical methods

The study aimed to enrolled 460 eligible women under the initial assumption that 95% would become pregnant within 1 year and 85% would be retained during follow-up. This sample size was based on a desired 80% power to detect a 135 g difference in mean birth weight between any two trial arms (threshold for statistical significance: 0.05; two-tailed tests) in a population with a 380 g SD in birth weight (based on a prior survey of 1200 births in the Cam Khe district in 2005).

In all analyses, categorical variables are presented as percentages, and continuous variables are presented as means and standard deviations. Continuous variables that were not normally distributed are presented as medians and interquartile ranges. Baseline maternal characteristics (i.e., education, occupation, living arrangements) and primary outcomes (i.e., maternal dietary intakes, maternal nutritional status, and birth outcomes) were compared across the assigned treatment groups using analysis of variance (normally distributed and continuous variables), chi-square (categorical variables), and Kruskal-Wallis (not normally distributed and continuous variables) tests. If the global test over the three treatment groups was statistically significant (p<0.05), pair-wise tests were performed (t-test, chi-square, or Mann-Whitney U, respectively). The primary outcomes of the trial were compared between trial arms without adjustment for baseline characteristics. Dietary recall data were compared using a model-based approach (generalized estimating equations, exchangeable working correlation structure, robust variance) to account for intra-participant variation, given that most participants had 2 recalls per time-point. Blood markers of maternal nutritional status were compared separately at each time point (i.e., baseline, mid-pregnancy, and late-pregnancy). The statistician was masked to trial group assignments when the statistical analyses were performed.

To examine the impact of the intervention on birth outcomes while adjusting for maternal characteristics, as well as to explore potential associations between maternal characteristics and birth outcomes, we fitted separate univariable and multivariable regression models for birth weight (hospital-measured and study-measured), birth length, and GWG. Adjustment variables included infant gender, gestational age, and maternal characteristics at baseline (i.e., age, education, occupation, living arrangement, height, body mass index <18.5, total energy and protein intakes) and during pregnancy (i.e., energy and protein intakes at 32 weeks of pregnancy). Covariables were checked for collinearity and variable inflation factors (mean: 1.99; largest: 3.92). Missing values (5% of covariable data) were multiply imputed (chained equations, formula-based method).

All statistical analyses were conducted using SPSS statistics 24 (IBM, Armonk, New York 10504–1722) and STATA version 14 (Statacorp, College Station, Texas 77845–4512); associations reaching p<0.05 were considered statistically significant.

## Results

### Sample characteristics

Two separate analyses were conducted for birth weight: one was based on the weights made at the District Hospital (N = 315) and the second was based on weights measured by study staff (N = 199) within a week of birth. In total, 317 infants had birth weight measured from at least one source. The women for whom study-measured weights were available had largely remained in contact with the study staff throughout pregnancy and complied with the assigned diet supplements (i.e., 191 of these 199 mothers had consumed at least 75% of the provided diet supplements and did not miss 10 or more consecutive meals). The hospital-measured weights included 118 women who stopped attending study visits and, therefore, were no longer receiving the food supplements. Thus, while not planned a priori, the two birth weight sources allowed for analyses that approximated intention-to-treat (hospital-measured) and per-protocol (study staff-measured) approaches.

### Participant recruitment and attrition

**Fig 1:**
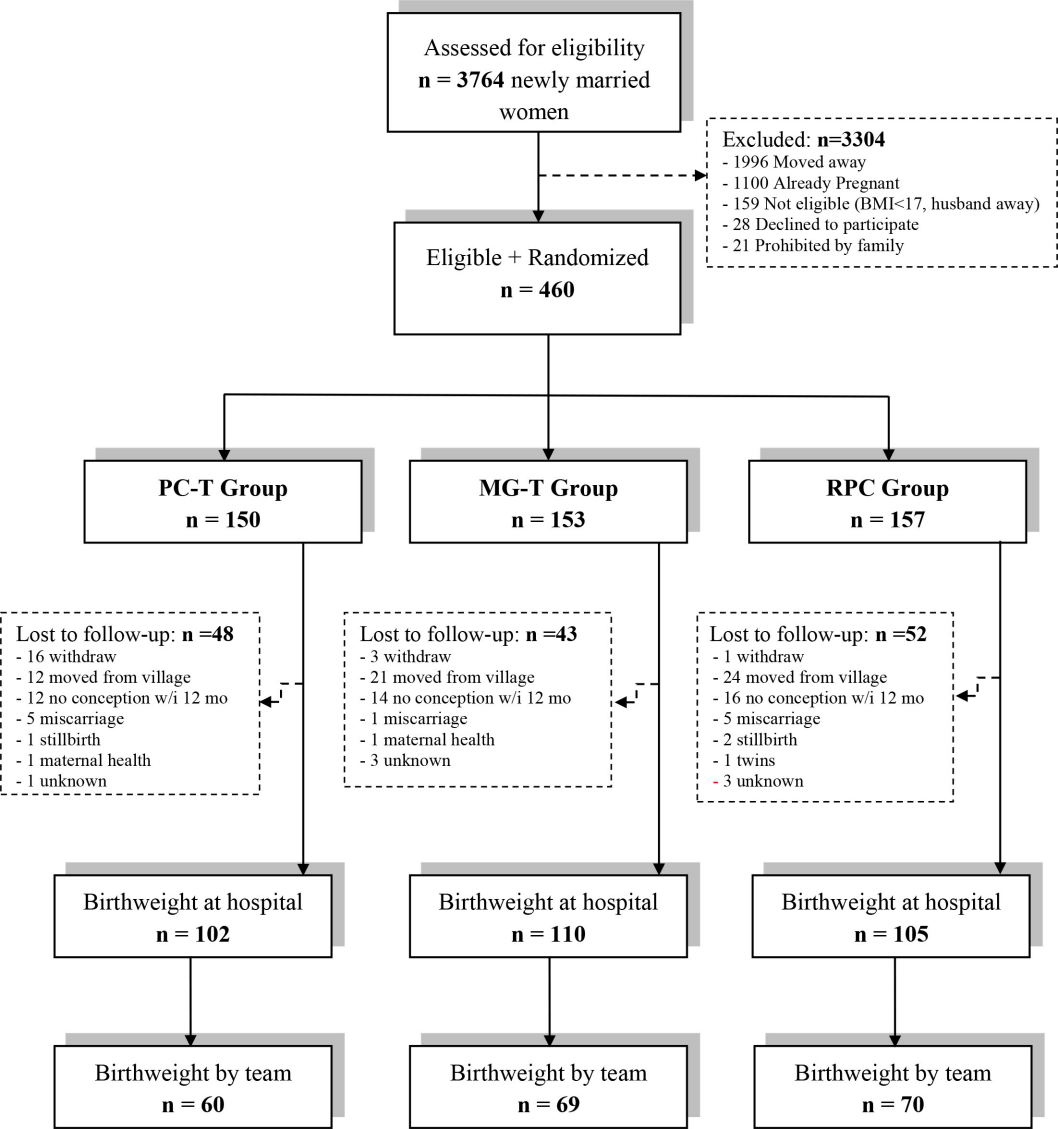
Flow diagram of participant progress throughout the VINAVAC study. PC-T, food supplement from pre-pregnancy to term; MG-T, food supplement from mid-pregnancy to term; RPC, routine perinatal care.

Of the 3,764 women who were initially identified as eligible for the study, 460 were enrolled **(Fig 1)**. More than 3,300 women (approximately 88% of the total) were excluded for the following reasons: 60% had plans to work outside of the study area, 33% were already pregnant or had a child previously, 5% did not meet the inclusion criterion of planning to have a baby within one year, did not live with their husband, or had a health problem, such as heart or kidney disease, and 2% declined to enroll without giving a reason. The 460 eligible women were randomly assigned to either the pre-conception to term (PC-T) food supplementation group, the mid-gestation to term (MG-T) group, or the routine prenatal care (RPC) group. Of those 460 women, 143 women (31%) did not finish the study for various reasons: they withdrew, moved from the village, did not conceive within 12 months, or had health problems that prevented continuing in the study (**Fig 1**). Of the 317 women who finished the study, as defined by having a recorded infant birth weight, the study team completed infant anthropometric measurements in 199 (63%) and hospital measures were available for 315 (99%). Actual losses to follow-up were higher than anticipated (although, the birth weight SD was lower), leaving the final sample with 84% power to detect a difference of 150 g between any two trial arms for the 315 hospital-measured infants and 64% power for the 199 study-measured infants.

### Baseline maternal characteristics

Characteristics of the participants are shown in **Table 2**. On average, the participants were 21 years old at enrollment; about 40% had education beyond middle school. As is typical in rural Vietnam, about 70% of the women lived with their husband’s parents and 78% worked as farmers. Approximately one-fourth of the women had a body mass index (BMI) below 18.5 kg/m^2^. Women dropping out of the study (**Table 3**) were less likely to live with their parents-in-law (59% vs. 71%) and to work as farmers (70% vs. 78%), had lower upper arm muscle area (21.8 cm^2^ vs. 23.4 cm^2^, p = 0.004) and greater arm fat areas (17.3 cm2 vs. 16.4 cm^2^) than the dropouts; however, only upper arm muscle area differences were statistically significant after accounting for multiple hypothesis tests (**Table 3**).

**Table 2:** Participant characteristics at baseline^1^.

	Participants (n = 317)	PC-T (n = 101)	MG-T (n = 110)	RPC (n = 106)
Age at random assignment, year^2^	21.4 [2.9]	21.5 [3.2]	21.1 [2.7]	21.6 [2.6]
Highest educational level^3^, %				
Elementary school	2.3	2.0	2.8	1.9
Middle school	56.8	54.1	58.9	57.1
High school	20.3	25.5	16.8	19.0
Occupational school or higher	20.6	18.4	21.5	21.9
Living arrangement^3^, %				
With parents-in-law	71.3	72.7	68.8	72.6
With husband, only	10.5	10.1	8.3	13.2
With parents	18.2	17.2	22.9	14.2
Work as farmers^3^, %	77.8	79.0	76.4	78.1
Anthropometry^2^				
Weight, kg	45.9 [4.8]	45.4 [4.6]	46.4 [5.0]	46.0 [4.9]
Height, cm	152.8 [5.1]	152.4 [5.0]	153.4 [4.6]	152.5 [5.6]
AMA, cm^2^	23.4 [4.8]	23.0 [4.4]	22.8 [4.7]	24.2 [5.2]
AFA, cm^2^	16.4 [4.4]	16.1 [4.0]	16.7 [4.7]	16.4 [4.4]
BMI, kg/m^2^	19.7 [1.8]	19.6 [1.6]	19.7 [1.8]	19.8 [1.9]
BMI < 18.5^3^, %	28.4	25.3	27.9	31.7

^1^ Sample sizes for each variable vary slightly because of item-specific missing data.

^2^ Values are means [SD]

^3^ Values are % of the total

PC-T, food supplement from pre-pregnancy to term, MG-T, food supplement from mid-pregnancy to term, RPC, routine perinatal care, AMA, arm muscle area; AFA, arm fat area; BMI, body mass index.

**Table 3:** Participant characteristics at baseline: Participants vs. drop-outs^1^.

	Completers (n = 317)	Drop-outs (n = 143)	p-value^4^
Age at random assignment, years^2^	21.4 [2.9]	21.9 [3.0]	0.06
Highest educational level^3^, %			0.16
Elementary school	2.3	2.9	
Middle school	56.8	47.1	
High school	20.3	20.3	
Occupational school or higher	20.6	29.7	
Living arrangement^3^, %			0.03
With parents-in-law	71.3	59.0	
With husband, only	10.5	16.5	
With parents	18.2	24.5	
Work as farmers^3^, %	77.8	69.8	0.07
Anthropometry^2^			
Weight, kg	45.9 [4.8]	45.9 [5.3]	0.91
Height, cm	152.8 [5.1]	152.4 [5.8]	0.55
AMA, cm^2^	23.4 [4.8]	21.8 [5.5]	0.004
AFA, cm^2^	16.4 [4.4]	17.3 [4.9]	0.05
BMI, kg/m^2^	19.7 [1.8]	19.7 [1.9]	0.73
BMI < 18.5^3^, %	28.4	24.5	0.39

^1^ Sample sizes for each variable vary slightly because of item-specific missing data.

^2^ Values are means [SD]

^3^ Values are % of the total

^4^ Comparisons between the completer and the drop-out groups: t-tests were used for comparing means of normally distributed continuous variables, the Mann-Whitney U test was used to compare means with a non-normal distribution for continuous variables, and a chi-square test was used to compare percentages of the population. Pair-wise differences statistically significant under the Benjamini-Hochberg procedure to maintain an overall false discovery rate ≤5% in bold.

AMA, arm muscle area; AFA, arm fat area; BMI, body mass index.

### Maternal diet and nutritional status

Maternal dietary intakes and nutritional status biomarkers of the women for whom such measures were available are shown in **Table 4**. At baseline, the nutrient intakes did not differ among the three groups. Protein, iron, zinc, vitamin A, folate, and vitamin B_12_ intakes were higher in the PC-T and MG-T groups compared to the RPC group (p<0.001) at mid-pregnancy. At late pregnancy, the nutrient intakes of the PC-T and MG-T groups exceeded the controls (p<0.001) except for energy (PC-T: 2111 kcal/day; MG-T: 2066 kcal/day; RPC: 1966 kcal/day, p = 0.17). The food-based supplement increased iron and folate intakes by about 60–80% and the protein and zinc intakes by about 30%. Vitamin A and B_12_ intakes were increased more than two-fold by the supplement.

**Table 4:** Maternal dietary intakes and nutritional status at baseline, mid and late pregnancy (N = 209)^1^.

	Baseline				Mid-Pregnancy				Late-Pregnancy				
	PC-T (n = 56)	MG-T (n = 68)	RPC (n = 73)	p-value ^2^	PC-T (n = 49)	MG-T (n = 57)	RPC (n = 63)	p-value ^2^	PC-T (n = 57)	MG-T (n = 57)	RPC (n = 66)		p-value ^2^
Weight, kg	45.6 [4.8]	46.5 [5.0]	46.3 [5.3]	0.58	46.8 [5.0]	47.4 [4.7]	46.7 [5.3]	0.74	53.4 [5.3]	53.7 [5.5]	53.5 [6.3]		0.96
**Nutrient Intakes**													
Energy intake, kcal/day	1741 [59]	1762 [43]	1792 [31]	0.70	2095 [65]	1902 [52]	1885 [55]	0.03	2111 [59]	2066 [52]	1966 [54]		0.17
Protein intake, g/day	70.3 [2.8]	70.6 [1.8]	72.6 [1.6]	0.64	95.3^a^ [3.5]	84.4^b^ [2.8]	75.4^c^ [2.3]	**<0.001**	95.5^a^ [2.6]	95.3^a^ [2.9]	75.8^b^ [2.5]		**<0.001**
Iron intake, mg/day	13.4 [0.8]	12.6 [0.4]	13.1 [0.4]	0.46	22.0^a^ [1.2]	19.9^a^ [1.3]	14.5^b^ [0.5]	**<0.001**	22.0^a^ [1.1]	21.6^a^ [1.1]	13.2^b^ [0.5]		**<0.001**
Zinc intake, mg/day	9.2 [0.4]	9.2 [0.2]	9.2 [0.2]	0.96	12.6^a^ [0.4]	11.8^a^ [0.5]	9.3^b^ [0.3]	**<0.001**	13.1^a^ [0.4]	12.8^a^ [0.4]	9.8^b^ [0.3]		**<0.001**
Vitamin A intake, μg/day	530 [37]	537 [52]	512 [35]	0.91	1977^a^ [195]	1730^a^ [174]	694^b^ [47]	**<0.001**	1973^a^ [150]	2012^a^ [154]	633^b^ [54]		**<0.001**
Folate intake, μg/day	327 [19]	327 [18]	305 [17]	0.59	536^a^ [33]	477^a^ [32]	350^b^ [31]	**<0.001**	539^a^ [28]	514^a^ [26]	287^b^ [18]		**<0.001**
Vitamin B_12_ intake, μg/day	2.1 [0.2]	2.3 [0.4]	2.1 [0.2]	0.89	6.4^a^ [0.7]	6.0^a^ [0.8]	3.0^b^ [0.7]	**<0.001**	6.6^a^ [0.7]	6.6^a^ [0.7]	2.2^b^ [0.2]		**<0.001**
**Nutritional Status Measurements**													
Hemoglobin, g/dL	12.9 [1.2]	12.9 [1.2]	12.8 [1.2]	0.88	11.6 [1.0]	11.7 [1.1]	11.6 [1.2]	0.66	11.5 [0.9]	11.8 [1.2]	11.6 [1.2]	0.27	
Hematocrit, %	40.3 [2.9]	40.2 [2.8]	40.1 [3.0]	0.90	35.5 [2.5]	35.2 [2.4]	35.1 [3.0]	0.78	35.3 [2.8]	35.9 [3.1]	35.3 [3.5]	0.58	
Anemia (Hb < 12 g/dL), %	25.5	19.7	20.9	0.73	12.8	15.8	15.4	0.90	13.7	15.0	12.9	0.94	
Plasma iron, μmol/L	16.9 [5.3]	17.1 [5.7]	17.2 [5.7]	0.52	19.8 [4.5]	20.0 [5.2]	20.2 [5.4]	0.93	15.9 [6.2]	17.5 [7.2]	16.1 [6.5]	0.38	
Ferritin (μg/L)	34.6 (21.0, 64.5)	57.4 (33.9, 93.0)	56.4 (30.9, 94.7)	0.05	47.5 (29.2, 91.2)	58.0 (28.3, 107.1)	69.4 (38.5, 119.6)	0.13	13.5 (2.0, 22.8)	10.4 (2.8, 21.1)	8.9 (2.1, 16.2)	0.83	
Serum tranferrin receptor (mg/L)	4.1 (3.0, 4.8)	3.6 (2.9, 4.6)	3.6 (3.0, 4.7)	0.56	2.9 (2.2, 3.7)	2.9 (2.2, 3.5)	2.8 (2.3, 3.4)	0.87	4.6 (3.6, 5.5)	4.1 (3.3, 5.3)	4.5 (3.6, 6.2)	0.52	
Plasma zinc, μmol/L	9.6 [1.3]	9.6 [1.4]	9.8 [1.5]	0.56	8.4 [1.3]	8.4 [1.2]	8.3 [1.3]	0.88	7.7 [1.0]	7.6 [1.2]	7.8 [1.1]	0.58	
Serum vitamin A, μmol/L	1.6 [0.4]	1.7 [0.4]	1.7 [0.4]	0.72	1.7 [0.3]	1.7 [0.4]	1.7 [0.4]	0.58	1.5 [0.3]	1.5 [0.4]	1.5 [0.4]	0.67	
Serum folate, nmol/L	16.9 (14.2–27.7)	18.7 (13.9, 27.2)	18.7 (14.9–23.3)	0.91	40.8 (28.9, 51.1)	39.8 (29.2, 47.0)	33.5 (24.7, 48.7)	0.20	36.8 (24.2, 52.0)	30.8 (17.9, 48.1)	34.1 (21.6–49.0)	0.42	
Serum cobalamin, pmol/L	662 (519, 820)	716 (584, 915)	673 (539, 831)	0.36	588 (457, 686)	557 (494, 697)	568 (451, 779)	0.93	427 (304, 560)	454 (357, 558)	401 (313, 526)	0.26	
Acute inflammation (CRP > 5 mg/L), %	1.9	6.3	0.0	0.08	6.5	3.5	7.7	0.63	5.6	9.8	3.2	0.31	
Chronic inflammation (AGP > 1 g/L), %	1.9	0.0	0.0	0.31	0.0	1.8	0.0	0.42	0.0	0.0	0.0	1.00	

^1^ Dietary recall nutritional intake values (top) are means [SEM], based on generalized estimating equation modeling to account for multiple dietary recalls per participant at each time point (working correlation structure: exchangeable). Sample size is based on N = 209 women who remained in contact with the study team throughout follow-up and with at least one dietary recall for at least one time point. Blood test nutritional status values (bottom) are means [SD], medians (25th, 75th percentiles), or percentages. PC-T, food supplement from pre-pregnancy to term, MG-T, food supplement from mid-pregnancy to term, RPC, routine perinatal care, CRP, C-reactive protein, AGP, α-1 acid glycoprotein.

^2^ Comparisons of the three treatment groups: model-based Wald test. If global (3-group) hypothesis test (dietary recall: Wald test; nutritional status: ANOVA, Kruskall Wallis, or chi-square) was significant under the Benjamini-Hochberg procedure to maintain an overall false discovery rate ≤5% (in bold), then any statistically significant pair-wise differences (p<0.05) between two groups are denoted with different superscript letters.

At baseline, serum ferritin levels **(Table 4)** were numerically lower in the PC-T group (35 μg/L) compared to the RPC group (56 μg/L), and by mid-pregnancy, the serum ferritin values of the PC-T (48 μg/L) and MG-T (58 μg/L) groups were lower than that of the RPC group (69 μg/L), but differences were not statistically significant. Serum ferritin levels were markedly lower at late pregnancy than at baseline in all three groups, and no group differences were evident. Despite the differences in intakes, biomarkers of the other iron status indicators as well as zinc, vitamin A, folate, and B_12_ did not differ among the three groups at any time point **(Table 4)**. Also, the incidence of anemia and inflammation among the three groups did not differ at baseline, mid or late pregnancy in the full cohort of 317 women or in the 199 women who complied with supplementation (see S1 Table).

Of the women who completed 24-h recalls, 23% took iron and folate supplements at 16 weeks gestation and 39% took supplements at 32 weeks. The distribution of supplement use did not differ among the three groups; it was 22% in the PC-T group, 21% in the MG-T group, and 26% in the RPC group (p = 0.54). Using micronutrient supplements at 16 and 32 weeks significantly increased serum folate levels at both time points (p<0.001), but taking those supplements was not associated with birth weight, birth length, or GWG.

### Pregnancy outcomes by intervention group

Birth outcomes of all of the women and of the three intervention groups are summarized in **Table 5**. Birth weight measured immediately after delivery by hospital staff averaged 3006 g, and it did not differ among the three diet groups. Birth weight measured by the study team during the first week of life were similar to the hospital measurements (2972 g), and it also did not differ among the three groups. The prevalence of low birth weight babies tended to be higher when evaluated by the study team rather than from hospital records, 8.0 vs 3.2%. This may reflect the practice of rounding birth weight to the nearest 100g by the hospital staff as well as the usual water loss by infants during the first few days of life causing a decrease in body weight [33]. Water loss is higher in infants weighing <2500 g that accounts for the higher prevalence of LBW infants measured by the study staff compared to the hospital staff [34]. Birth length and infant mid-upper arm circumference (MUAC), measured by the study team, did not differ among the three groups. Infants in the RPC group had a numerically larger head circumference than that of infants in the two food intervention groups, but not statistically significantly different. The mean head circumference for all groups was below the 50th percentile, and the difference is likely within the error of the measurement [26]. In the entire cohort, 10.6% of the babies were preterm, i.e., were born before 37 weeks of gestation, and 16.1% were small for gestational age, i.e., had birth weights <10^th^ percentile for the gestational age. Maternal gestational weight gain averaged 7.4 kg in the entire cohort during the first 32 weeks of gestation and it did not differ among the treatment groups. On average, the women gained 0.40 kg/week between 16 and 32 weeks of pregnancy.

**Table 5:** Comparison of birth outcomes by treatment group^1^.

All participants (n = 317)^2^		PC-T (n = 101)	MG-T (n = 110)	RPC (n = 106)	p-value
Male, %	46.1	48.5	46.4	43.4	0.76
Birth weight (hospital), g	3006 [367]	2983 [345]	2991 [421]	3043 [326]	0.44
Birth weight (study staff), g	2972 [349]	2938 [292]	2951 [395]	3021 [346]	0.33
Low birth weight (hospital) (< 2500 g), %	3.2	3.0	5.5	1.0	0.17
Low birth weight (study staff) (< 2500 g), %	8.0	6.7	11.6	5.6	0.39
Birth length, cm	49.1 [1.8]	49.0 [1.6]	48.9 [2.2]	49.2 [1.5]	0.68
Infant MUAC, cm	10.3 [1.0]	10.2 [1.0]	10.3 [1.1]	10.3 [0.8]	0.74
Infant head circumference, cm	33.9 [1.6]	33.6 [1.6]	33.6 [1.4]	34.4 [1.6]	0.01
Preterm birth, %^4^	10.6	13.5	13.3	5.7	0.17
Gestational age, wk	39.1 [2.0]	39.0 [2.0]	38.9 [2.1]	39.5 [1.9]	0.14
Gestational weight gain, kg^5^	7.4 [3.6]	7.6 [3.4]	7.5 [3.0]	7.3 [4.2]	0.94
Weight small for gestational age, %	16.1	13.7	20.7	13.8	0.38
Weight gain between 16 and 32 weeks, kg/week	0.40 [0.16]	0.41 [0.15]	0.38 [0.14]	0.42 [0.18]	0.36

^1^ Values are means [SD], or percentages. Sample sizes for each variable vary slightly because of item-specific missing data. PC-T, food supplement from pre-pregnancy to term, MG-T, food supplement from mid-pregnancy to term, RPC, routine perinatal care

^2^ Sample smaller than 317 (up to n = 199) for measurements completed by study staff.

^3^ The ANOVA test was used to compare the means of the three treatment groups, and chi-square test was used to compare the percentage differences among the three groups. None of the comparisons are statistically significant under the Benjamini-Hochberg procedure to maintain an overall false discovery rate ≤5%.

^4^ Pre-term birth was defined as a birth before 37 weeks of pregnancy.

^5^ Gestational weight gain was the weight gained during the first 32 weeks of pregnancy.

### Pregnancy outcomes in the entire study cohort

After adjustment for infant and maternal variables, there were no statistically significant associations between the food supplement intervention and birth outcomes. However, infant gestational age at delivery as measured at the hospital and by study staff was positively associated with birth weight and birth length (**Tables 6 and 7**). In our multivariable model, for each standard deviation increase in gestation age (approximately 2 weeks), there was a statistically significant increase in team-measured birth weight (129 g) and birth length (0.5 cm), but not in hospital-measured birth weight after accounting for multiple hypothesis tests.

**Table 6:** Birth weight outcomes by trial group and maternal and infant characteristics.

	Hospital-Measured Birth Weight, g (N = 315)				Study-Measured Birth Weight, g (N = 199)			
	Univariable Model		Multivariable Model		Univariable Model		Multivariable Model	
	Unadjusted Difference, g (95% CI)	p-value	Adjusted Difference, g (95% CI)	p-value	Unadjusted Difference, g (95% CI)	p-value	Adjusted Difference, g (95% CI)	p-value
Infant Characteristics								
RPC	reference		reference		reference		reference	
MG-T	-51 (-150, 47)	0.31	-44 (-154, 66)	0.43	-71 (-187, 45)	0.23	-17 (-142, 108)	0.79
PC-T	-60 (-161, 41)	0.24	-50 (-168, 68)	0.41	-83 (-204, 38)	0.18	-22 (-154, 109)	0.74
Female infant	-76 (-157, 6)	0.07	-74 (-157, 9)	0.08	-48 (-146, 50)	0.34	-56 (-152, 39)	0.25
Gestational age, (std.)	59 (13, 103)	0.01	48 (2, 94)	0.04	126 (78, 174)	**<0.001**	129 (80, 179)	**<0.001**
Maternal Characteristics								
Age, years	9 (-5, 24)	0.20	7 (-9, 23)	0.37	6 (-11, 24)	0.48	-4 (-23, 14)	0.66
Height, (std.)	47 (3, 90)	0.03	42 (-4, 88)	0.07	59 (9, 110)	0.02	51 (-1, 104)	0.05
BMI < 18.5	-20 (-113, 72)	0.67	-21 (-112, 71)	0.66	-10 (-122, 101)	0.85	-17 (-122, 89)	0.75
Baseline energy intake, (std.)	3 (-42, 47)	0.90	29 (-57, 114)	0.51	50 (-3, 104)	0.06	91 (-7, 188)	0.07
32-week energy intake, (std.)	7 (-46, 60)	0.79	-2 (-82, 79)	0.96	31 (-22, 84)	0.29	46 (-42, 134)	0.30
Baseline protein intake, (std.)	-9 (-53, 36)	0.70	-41 (-126, 44)	0.35	23 (-29, 75)	0.39	-66 (-160, 28)	0.17
32-week protein intake, (std.)	-1 (-53, 51)	0.98	-2 (-91, 88)	0.97	6 (-46.5, 59)	0.81	-32 (-131, 67)	0.52
Farmer occupation	-12 (-110, 86)	0.81	38 (-87, 162)	0.55	2 (-109,113)	0.97	19 (-113, 152)	0.77
Educational attainment								
< High school	reference		reference		reference		reference	
High school	73 (-32, 177)	0.17	58 (-54, 171)	0.31	28 (-95, 151)	0.65	37 (-92, 166)	0.58
Occupational school or above	88 (-15, 192)	0.09	107 (-29, 243)	0.12	69 (-52, 190)	0.26	123 (-26, 273)	0.11
Lives with family in-law	35 (-55, 124)	0.45	22 (-72, 117)	0.64	63 (-44, 170)	0.25	30 (-80, 140)	0.59

Abbreviations: RPC = routine prenatal care; MG-T = food supplement from midgestation to term; PC-T = food supplement from preconception to term; BMI = body mass index; CI = confidence interval; std. = standardized: one unit corresponds to one standard deviation of the observed variable

Statistical significance (in bold) calculated under the Benjamini-Hochberg procedure to maintain an overall false discovery rate ≤5%.

**Table 7:** Birth length and gestational weight gain outcomes by trial group and maternal and infant characteristics.

	Study-Measured Birth Length, cm (N = 195)				Study-Measured Gestational Weight Gain, kg (N = 197)			
	Univariable Model		Multivariable Model		Univariable Model		Multivariable Model	
	Unadjusted Difference, cm (95% CI)	p-value	Adjusted Difference, cm (95% CI)	p-value	Unadjusted Difference, cm (95% CI)	p-value	Adjusted Difference, cm (95% CI)	p-value
***Infant Characteristics***								
RPC	reference		reference		reference		reference	
MG-T	-0.3 (-0.9, 0.3)	0.39	0.0 (-0.7, 0.7)	0.99	0.1 (-1.1, 1.3)	0.85	-0.1 (-1.3, 1.0)	0.83
PC-T	-0.2 (-0.8,0.5)	0.57	0.0 (-0.7, 0.8)	0.93	0.2 (-1.0, 1.5)	0.72	-0.1 (-1.4, 1.1)	0.85
Female infant	-0.6 (-1.1,0.1)	0.02	-0.6 (-1.1,-0.1)	0.03	0.7 (-0.3, 1.7)	0.16	0.8 (-0.1, 1.7)	0.07
Gestational age, (std.)	0.5 (0.2, 0.7)	**<0.001**	0.5 (0.2, 0.8)	**<0.001**	-0.1 (-0.7, 0.5)	0.79	0.0 (-0.5, 0.6)	0.90
***Maternal Characteristics***								
Age, years	0.0 (0.0, 0.1)	0.35	0.0 (-0.1, 0.1)	0.54	0.2 (0.0, 0.3)	0.07	0.0 (-0.2, 0.2)	0.88
Height, (std.)	0.3 (0.0, 0.6)	0.02	0.3 (0.0, 0.6)	0.06	0.9 (0.4, 1.4)	**<0.001**	0.7 (0.2, 1.2)	0.01
BMI < 18.5	-0.2 (-0.8,0.4)	0.54	-0.2 (-0.8, 0.4)	0.53	2.3 (1.2, 3.4)	**<0.001**	2.3 (1.3, 3.4)	**<0.001**
Baseline energy intake, (std.)	0.1 (-0.2,0.4)	0.44	0.1 (-0.4, 0.7)	0.63	0.1 (-0.4, 0.7)	0.65	-0.4 (-1.3, 0.5)	0.38
32-week energy intake, (std.)	0.0 (-0.3,0.2)	0.76	0.0 (-0.5, 0.5)	0.95	0.4 (-0.1, 0.9)	0.14	0.0 (-0.9, 0.8)	0.95
Baseline protein intake, (std.)	0.1 (-0.2,0.3)	0.69	-0.1 (-0.6, 0.4)	0.73	0.4 (-0.1, 0.9)	0.15	0.6 (-0.3, 1.5)	0.20
32-week protein intake, (std.)	0.0 (-0.3,0.2)	0.76	-0.1 (-0.6, 0.5)	0.84	0.5 (0.0, 1.0)	0.04	0.4 (-0.5, 1.3)	0.39
Farmer Occupation	0.0 (-0.5,0.6)	0.89	-0.2 (-0.9, 0.5)	0.59	-3.0 (-4.0,-1.9)	**<0.001**	-1.4 (-2.7, -0.1)	0.03
**Educational attainment**								
< High school	reference		reference		reference		reference	
High school	0.0 (-0.6,0.7)	0.93	-0.1 (-0.8, 0.6)	0.79	2.8 (1.6, 4.0)	**<0.001**	1.9 (0.6, 3.1)	**<0.001**
Occupational school or above	-0.2 (-0.8,0.4)	0.55	-0.2 (-1.0, 0.7)	0.67	2.8 (1.7, 3.8)	**<0.001**	1.5 (0.1, 2.9)	0.03
Lives with family in-law	0.5 (-0.1,1.0)	0.11	0.3 (-0.3, 0.9)	0.34	0.2 (-1.9, 1.3)	0.77	-0.1 (-1.1, 1.0)	0.91

Abbreviations: RPC = routine prenatal care; MG-T = food supplement from midgestation to term; PC-T = food supplement from preconception to term; BMI = body mass index; CI = confidence interval; std. = standardized: one unit corresponds to one standard deviation of the observed variable

Statistical significance (in bold) calculated under the Benjamini-Hochberg procedure to maintain an overall false discovery rate ≤5%.

Maternal height was positively associated with gestational weight gain (each 5 cm of greater height associated with 0.9 kg of weight gain, unadjusted model) (**Table 7**). A low pre-pregnancy BMI (< 18.5) was also associated with greater GWG, with lower BMI women gaining about 2.3 kg more than higher BMI women after adjustment for other variables. Several socioeconomic variables were associated with GWG. Working as a farmer compared to other professions was associated with lower GWG (-3.0 kg, unadjusted; 1.4 kg, adjusted), while higher educational attainment compared to below high school level was associated with greater GWG (high school graduate: 1.9 kg; higher occupation training: 1.5 kg, adjusted). GWG gain itself was positively associated with hospital-measured birth weight (not shown in tables). For each standard deviation of greater GWG (approximately 3.6 kg), the corresponding increase for hospital-measured birth weight was 89 g (95% CI: 34 g, 143 g; p = 0.002; unadjusted) and 51 g (95% CI: -14 g, 116 g; p = 0.12; adjusted); for study-measured birth weight the increase was 42 g (95% CI: -8 g, 93 g; p = 0.10; unadjusted) and 25 g (95% CI: -36 g, 86 g; p = 0.42; adjusted).

## Discussion

In this randomized-controlled trial, provision of a small (190 kcal/d) nutrient-rich, food-based supplement five days/week from prior to conception to term did not alter pregnancy outcomes as measured by birth weight, length, the prevalence of preterm births, or the occurrence of small-for-gestational babies. Although the supplement significantly increased energy, vitamin A, B_12_, folate, zinc, and iron intakes prior to and during pregnancy, circulating biomarkers of those nutrients did not differ among the three groups at either mid or late pregnancy even though the nutrient-dense food supplement increased the nutrient intakes by 30 to 290%. However, usual intakes of the target nutrients, i.e., iron, zinc, vitamin A, folate, and vitamin B_12,_ either exceeded (i.e., iron and zinc) or were equal to the Estimated Average Requirements (i.e., vitamin A, folate, and B_12_) [23]. Absence of an effect on circulating levels of the targeted nutrients, along with a smaller than anticipated final sample size due to economic factors in Vietnam that encouraged women to seek employment distant from the study area, limited our ability to detect any effect of the intervention on newborn size. Nevertheless, the study provides novel data on pregnancy outcomes in rural Vietnamese women who are involved in demanding physical work throughout gestation.

In a study of non-pregnant, rural Vietnamese women done 10 years earlier, about 15% were anemic and about 40% had a BMI below 18.5 [15]. Those data led to the hypothesis for this study that a nutrient-dense, food-based supplement given to Vietnamese women prior to conception would improve their weight and iron status at conception, which in turn would improve pregnancy outcomes. This theory was supported by human and animal study data showing that improving maternal nutritional status prior to conception modulated placental metabolism and improved fetal growth and postnatal health [35, 36]. Several human studies showed that providing nutrient supplements to women between pregnancies improved birth weight and length and reduced maternal anemia in a subsequent pregnancy more than if the supplements were only given during pregnancy [5, 37]. In addition to our study, two other groups with much larger sample sizes failed to find a positive effect of pre-pregnancy supplements on birth outcomes. A large trial of over 7300 women from four countries (Guatemala, India, Pakistan, and the Democratic Republic of the Congo) failed to show that the length-for-age z-score of infants born to mothers receiving a lipid-based micronutrient supplement ≥ 3 months before conception differed from those who got the supplement late in the first trimester [38]. Another trial in Vietnam also failed to find a positive effect of a multiple micronutrient supplement taken prior to conception on birth outcomes [39]. These diverse outcomes of the effect of nutrient supplementation prior to conception are likely related to the initial nutritional status of the women. For example, a study in Mumbai, India showed that dietary energy and micronutrients may be partitioned differently between the mother and fetus in underweight compared to normal weight mothers [6]. Provision of a nutrient-rich snack from prior to conception to term increased birth weights only if the mother was not underweight and had received the nutrient supplements for at least three months prior to conception. As suggested earlier [37], there appears to be a biological trade-off between maintenance of maternal nutrition and increasing fetal size. Studies in animal models show that maternal undernutrition at conception influences placental structure and function that ultimately influences the fetal nutrient supply and metabolism [36]. Data from this study, along with that from others, supports the concept that maternal parenting begins prior to conception through nutrition [35].

Although our nutrient-rich, food-based supplement provided about half of the iron and zinc RDA and more than doubled the vitamin A, folate, and B_12_ recommended intakes, circulating concentrations of these nutrients did not differ among the three groups at either mid or late pregnancy. There are several reasons why the increased intake of animal source foods with good nutrient bioavailability failed to increase serum nutrient levels. One explanation is the lower nutrient absorption when it is consumed either from or with food compared to taking a supplement in the fasting state. Several studies have shown that more zinc is absorbed from a supplement taken without food rather than with a meal [40, 41]. Also, the usual pregnancy plasma volume increase of about 40% could have obviated any changes in serum nutrient levels even with an increase in absorption [42]. Finally, the intake of a relatively good diet by our participants prior to and during pregnancy may have prevented an effect of the intervention on maternal nutritional status.

In a secondary data analysis, we studied the maternal characteristics associated with birth outcomes in the entire cohort of 317 women **(Tables 5 and 6)**. In our model, birth weight increased about 50 g and length increased about 0.3 cm (p<0.05) for every cm increase in maternal height. These positive effects of maternal height on birth weight and length maybe related to the higher gestational weight gains (about 0.8 kg) by the taller women (p<0.01). However, the anatomical factors of taller women, i.e. a larger uterus or pelvis, may allow more fetal growth [43, 44]. An increase in maternal height may also reflect a better diet or a higher socio-economic status that reduced the risk of a low birth weight baby. This explanation is supported by the fact that maternal height and gestational weight gain were positively associated in our study. For every standard deviation increase in height, GWG increased by 0.9 kg (p<0.001). These data, as well as others reported in the literature, suggest that short maternal stature could be an easily measurable risk of increased preterm birth or low birth weights [45, 46].

Although the prevalence of underweight women has declined in Vietnam over the past 10 years, the median birth weight is unchanged [47]. The lower birth weight per unit weight gain among the Vietnamese women suggests that more of the mother’s energy intake was used for maternal metabolic and physical work rather than fetal growth. For example, working as a farmer reduced GWG 1.5–3.0 kg. But, working as a farmer did not have a negative effect of birth weight or length. These results differ from the results of a survey of birth weights among farming mothers in Northern Vietnam in 1992–3 [48]. Twenty years ago, farming mothers were twice as likely to have a low birth weight baby that was probably linked to an insufficient food supply. Today, farming is a risk factor for lower GWGs, but without significantly affecting fetal growth. This suggests that the food supply of farming pregnant women has improved and/or the severity of physical activity has declined in the past 20 years.

Among the three treatment arms, the mean birth weight in the routine prenatal care (control) group was about 68 g higher than that in the PC-T group. Of the maternal characteristics measured, only the baseline arm muscle area differed between the RPC and the supplemented groups; it tended to be higher in the RPC group (24.3 vs 22.8, p = 0.06). Others have reported that pregnant women in lower income countries with larger upper arm muscle areas tend to have heavier infants [49, 50]. This increase in the arm circumference may reflect an increase in maternal protein reserves (muscle) that better supports fetal growth.

A serious limitation of this study was the high rate of attrition prior to conception and during the first trimester of pregnancy that significantly reduced our power to find an effect of the food supplement on newborn size. Nearly 90% of the eligible women did not qualify because they were already pregnant, had plans to move out of the area, did not meet our inclusion criteria, declined to participate, or were prohibited from participating by family members. Of the 460 women who consented, only 68% completed the study, potentially reflecting the participant burden of the coming to the feeding site five days/week. Since the drop-outs were slightly less likely to work as farmers, they were probably better economically and, therefore, more likely to take jobs outside the study area. However, it is important to note that the direction of the observed effect did not favor the intervention. Another limitation was the lack of full compliance with the study. Many women did not eat all of the scheduled supplemental meals. However, among the 199 women with team-measured infant anthropometric values, the vast majority (191) had consumed at least 75% of the provided supplements, and no main effect of the intervention was observed. Although not planned, this “complier” sample served as an estimate of the efficacy of the intervention whereas the larger sample of 315 women, who had hospital-measured birthweights, was more analogous to an effectiveness study population (i.e., effect among those assigned to the intervention regardless of compliance). Both samples resulted in the same conclusion, no main effect on birth weight.

In conclusion, a small nutrient-dense supplement provided to rural Vietnamese women prior to conception did not improve birth outcomes. The study is unique in that nearly 80% of the participants worked as farmers and gained only about 7.5 kg during their pregnancies. Performing heavy agricultural work during pregnancy, which is not uncommon in low or middle income countries, has been associated with lower birth weights [51]. Future nutritional interventions need to target women performing heavy physical work during pregnancy. It is likely that the heavy work performed by our participants offset any potential benefits of the low energy, nutrient-rich food supplement on fetal growth. Our population of farming women would likely have benefitted more from a food supplement providing more energy.

## Supporting information

S1 Checklist CONSORT 2010 checklist of information to include when reporting a randomised trial*. (DOCX)

S1 File Vietnam birth outcomes. (CSV)

S1 Table Maternal dietary intakes and nutritional status at baseline, mid and late pregnancy of 199 women with a team-measured birth weight^1^. (DOCX)

S1 Protocol Application for study review. (DOC)

## Abbreviations

AFA: arm fat area
AGP: α-1-acid glycoprotein
AMA: arm muscle area
BMI: body mass index
CRP: C-reactive protein
GWG: gestational weight gain
LBW: low birth weight
MG-T: mid-gestation to term
MUAC: mid-upper arm circumference
PC-T: pre-conception to term
RDA: Recommended dietary allowance
RPC: Routine prenatal care
