# Supplementary material for: Effect of a nutrient-rich, food-based supplement given to rural Vietnamese mothers prior to and/or during pregnancy on birth outcomes: A randomized controlled trial
Source: PLoS One. 2020 May 29;15(5):e0232197. doi: 10.1371/journal.pone.0232197 (PMC7259625; doi:10.1371/journal.pone.0232197)
Supplement: S1 Table — (DOCX) [file pone.0232197.s003.docx]

**Appendix Table 1.** **Maternal dietary intakes and nutritional status at baseline, mid and late pregnancy of 199 women with a team-measured birth weight^1^**.

|  | **Baseline** | | | | **Mid-Pregnancy** | | | | **Late-Pregnancy** | | | |  | |  |
| --- | --- | --- | --- | --- | --- | --- | --- | --- | --- | --- | --- | --- | --- | --- | --- |
|  | **PC-T** (n=58) | **MG-T** (n=69) | **RPC** (n=69) | **p^2^** | **PC-T** (n=58) | **MG-T** (n=69) | **RPC** (n=69) | **p^2^** | **PC-T** (n=58) | **MG-T** (n=69) | **RPC** (n=69) | | **p^2^** | |  |
| Weight, kg |  |  |  |  | 46.8 ± 5.0 | 47.4 ± 4.7 | 46.7 ± 5.3 | 0.74 | 53.4 ± 5.3 | 53.7 ± 5.5 | 53.5 ± 6.3 | | 0.96 | |  |
| **Nutrient Intakes** |  |  |  |  |  |  |  |  |  |  |  | |  | |  |
| Energy intake, kcal/day | 1748 ± 410 | 1819 ± 365 | 1791 ± 267 | 0.56 | 2100 ± 483^a^ | 1922 ± 614^b^ | 1943 ± 448^b^ | 0.10 | 2102 ± 453 | 2073 ± 397 | 1943 ± 450 | | 0.12 | |  |
| Protein intake, g/day | 69.7 ± 20.9 | 72.7 ± 15.0 | 71.8 ± 13.6 | 0.62 | 93.6 ± 24.9^a^ | 85.8 ± 20.5^b^ | 76.4 ± 18.6^b^ | **0.00** | 95.0 ± 20.1^a^ | 94.7 ± 22.5^a^ | 75.5 ± 20.2^b^ | | **0.00** | |  |
| Iron intake, mg/day | 13.2 ± 5.9 | 13.0 ± 3.1 | 13.0 ± 2.9 | 0.97 | 21.9 ± 8.9^a^ | 20.1 ± 9.7^b^ | 14.7 ± 3.5^b^ | **0.00** | 21.8 ± 8.4^a^ | 20.4 ± 7.8^a^ | 12.0 ± 3.8^b^ | | **0.00** | |  |
| Zinc intake, mg/day | 9.3 ± 2.8 | 9.3 ± 2.1 | 9.1 ± 1.4 | 0.79 | 12.3 ± 3.0^a^ | 11.8 ± 3.3^b^ | 9.6 ± 2.7^b^ | **0.00** | 13.1 ± 3.2^a^ | 12.5 ± 3.2^a^ | 9.6 ± 2.7^b^ | | **0.00** | |  |
| Vitamin A intake, μg/day | 483 ± 267 | 541 ± 426 | 520 ± 301 | 0.68 | 1880 ± 1332^a^ | 1741 ± 1276^b^ | 696 ± 365^b^ | **0.00** | 1931 ± 1099^a^ | 1858 ± 1143^a^ | 639 ± 441^b^ | | **0.00** | |  |
| Folate intake, μg/day | 306 ± 128 | 328 ± 159 | 316 ± 156 | 0.74 | 512 ± 224^a^ | 474 ± 242^b^ | 359 ± 248^b^ | **0.01** | 514 ± 211^a^ | 485 ± 198^a^ | 287 ± 157^b^ | | **0.00** | |  |
| Vitamin B_12_ intake, μg/day | 1.8 (0.5, 2.6) | 1.8 (0.6, 2.8) | 1.8 (1.1, 2.8) | 0.75 | 4.6 (1.6, 8.3)^a^ | 3.1 (1.5, 8.9)^b^ | 1.9 (0.9, 3.7)^b^ | **0.00** | 6.8 (1.4, 10.5)^a^ | 5.5 (1.5, 8.4)^a^ | 1.8 (0.7, 3.0)^b^ | | **0.00** | |  |
| **Nutritional Status Measurements** | | | | | | | | | | | | | | | |
| Hemoglobin, g/dL | 12.9 ± 1.2 | 12.9 ± 1.2 | 12.8 ± 1.2 | 0.88 | 11.6 ± 1.0 | 11.7 ± 1.1 | 11.6 ± 1.2 | 0.66 | 11.5 ± 0.9 | 11.8 ± 1.2 | 11.6 ± 1.2 | 0.27 | | |  |
| Hematocrit, % | 40.3 ± 2.9 | 40.2 ± 2.8 | 40.1 ± 3.01 | 0.90 | 35.5 ± 2.5 | 35.2 ± 2.4 | 35.1 ± 3.0 | 0.78 | 35.3 ± 2.8 | 35.9 ± 3.1 | 35.3 ± 3.5 | 0.58 | | |  |
| Anemia (Hb < 12 g/dL), % | 25.5 | 19.7 | 20.9 | 0.73 | 12.8 | 15.8 | 15.4 | 0.90 | 13.7 | 15.0 | 12.9 | 0.94 | |  |  |
| Plasma iron, μmol/L | 16.9 ± 5.3 | 17.1 ± 5.7 | 17.2 ± 5.7 | 0.52 | 19.8 ± 4.5 | 20.0 ± 5.2 | 20.2 ± 5.4 | 0.93 | 15.9 ± 6.2 | 17.5 ± 7.2 | 16.1 ± 6.5 | 0.38 | |  |  |
| Ferritin (µg/L) | 34.6  (21.0, 64.5)^a^ | 57.4  (33.9, 93.0) | 56.4  (30.9, 94.7)^a^ | 0.05 | 47.5  (29.2, 91.2) | 58.0  (28.3, 107.1) | 69.4  (38.5, 119.6) | 0.13 | 13.5  (2.0, 22.8) | 10.4  (2.8, 21.1) | 8.9  (2.1, 16.2) | 0.83 | |  |  |
| Serum tranferrin receptor (mg/L) | 4.1  (3.0, 4.8) | 3.6 (2.9, 4.6) | 3.6  (3.0, 4.7) | 0.56 | 2.9  (2.2, 3.7) | 2.9  (2.2, 3.5) | 2.8  (2.3, 3.4) | 0.87 | 4.6  (3.6, 5.5) | 4.1  (3.3, 5.3) | 4.5  (3.6, 6.2) | 0.52 | |  |  |
| Plasma zinc, μmol/L | 9.6 ± 1.3 | 9.6 ± 1.4 | 9.8 ± 1.5 | 0.56 | 8.4 ± 1.3 | 8.4 ± 1.2 | 8.3 ± 1.3 | 0.88 | 7.7 ± 1.0 | 7.6 ± 1.2 | 7.8 ± 1.1 | 0.58 | |  |  |
| Serum vitamin A, μmol/L | 1.6 ± 0.4 | 1.7 ± 0.4 | 1.7 ± 0.4 | 0.72 | 1.7 ± 0.3 | 1.7 ± 0.4 | 1.7 ± 0.4 | 0.58 | 1.5 ± 0.3 | 1.5 ± 0.4 | 1.5 ± 0.4 | 0.67 | |  |  |
| Serum folate, nmol/L | 16.9  (14.2-27.7) | 18.7  (13.9, 27.2) | 18.7  (14.9-23.3) | 0.91 | 40.8  (28.9, 51.1) | 39.8  (29.2, 47.0) | 33.5  (24.7, 48.7) | 0.20 | 36.8  (24.2, 52.0) | 30.8  (17.9, 48.1) | 34.1  (21.6-49.0) | 0.42 | |  |  |
| Serum cobalamin, pmol/L | 662  (519, 820) | 716  (584, 915) | 673  (539, 831) | 0.36 | 588  (457, 686) | 557  (494, 697) | 568  (451, 779) | 0.93 | 427  (304, 560) | 454  (357, 558) | 401  (313, 526) | 0.26 | |  |  |
| Acute inflammation (CRP > 5 mg/L), % | 1.9 | 6.3 | 0.0 | 0.08 | 6.5 | 3.5 | 7.7 | 0.63 | 5.6 | 9.8 | 3.2 | 0.31 | |  |  |
| Chronic inflammation (AGP > 1 g/L), % | 1.9 | 0.0 | 0.0 | 0.31 | 0.0 | 1.8 | 0.0 | 0.42 | 0.0 | 0.0 | 0.0 | ns | |  |  |

^1^ Values are means ± SDs, medians (25^th^, 75^th^ percentiles), or percentages of the total population. Sample sizes for each variable vary slightly because of item-specific missing data. PC-T, food supplement from pre-pregnancy to term, MG-T, food supplement from mid-pregnancy to term, RPC, routine perinatal care, CRP, C-reactive protein, AGP, α-1 acid glycoprotein.

^2^ Comparisons of the three treatment groups: an ANOVA test was used to compare means of normally distributed continuous variables, the Kruskal Wallis test for comparing means with a non-normal distribution for continuous variables and, and a chi-square test was used to compare percentages of the population. Statistically significant differences (p<0.05) between any two groups are denoted with different superscript letters
