# Supplementary material for: Effect of a nutrient-rich, food-based supplement given to rural Vietnamese mothers prior to and/or during pregnancy on birth outcomes: A randomized controlled trial
Source: PLoS One. 2020 May 29;15(5):e0232197. doi: 10.1371/journal.pone.0232197 (PMC7259625; doi:10.1371/journal.pone.0232197)
Supplement: S1 Protocol — (DOC) [file pone.0232197.s004.doc]

# Institutional Review Board

# Application for Study Review

| Study Information | |
| --- | --- |
| **IRB Number:** | 2010-068 (assigned by the IRB staff) |
| **Protocol Title:** | Effect of Animal Source Food Supplement Prior to and During Pregnancy on Birth Weight and Prematurity in Rural Vietnam |
| **Principal Investigator:** | Janet King, R.D., Ph.D. |
| Department: | CHORI |
| Address (if outside CHRCO): |  |
| Phone: | (510) 450-7939 |
| Fax: | (     )      - |
| E-mail: | jking@chori.org |
| **Principal Investigator:** | Deborah Dean, M.D., M.P.H. |
| Department: | CHORI |
| Address (if outside CHRCO): |  |
| Phone: | (510) 450-7655 |
| Fax: | (510) 450-7910 |
| E-mail: | ddean@chori.org |
| **Principle Investigator:** | Tu Ngu, M.D., Ph.D. |
| Department: | National Institute of Nutrition |
| Address (if outside CHRCO): | 48B Tang Bat Ho St., Hanoi, Vietnam |
| Phone: | (8443) 971-3089 |
| Fax: | (8443) 971-7885 |
| E-mail: | tungu.nin@gmail.com |
| **Study Coordinator:** | Andrew Hall, Ph.D. |
| Phone: | (8443) 971-3089 |
| Fax: | (     )      - |
| E-mail: | andrew.hall@fulbrightmail.org |
| **Primary Contact Person:** | Janet King |

| Funding | | | |
| --- | --- | --- | --- |
| Federal Pharmaceutical Company Private Foundation Internal Grant Program  Industry  Children's Oncology Group (COG)  Other, Specify: | | | |
| **Funding Source:** | | Thrasher Research Fund; the Nestle Foundation | |
| **Contract or Grant Title:** | | Effect of Animal Source Food Supplement Prior to and During Pregnancy on Birth Weight and Prematurity in Rural Vietnam | |
| **Contract or Grant #:** | |  | |
| **Address of Sponsor:** | 15 W. South Temple Street, Suite 1650; Place de la Gare 4 - PO Box 581 - CH-1001 | | |
|  | Salt Lake City, UT 84101; Lausanne, Switzerland | | |
| **Contact Person:** | | R. Justin Brown, MPH; Paolo Sutter, MD | |
| **Phone:** | | | (801) 240-2838; +41 21 320 33 51 |
| **E-mail:** | | | [BrownRJ@thrasherresearch.org](mailto:BrownRJ@thrasherresearch.org); nf@nestlefoundation.org |

| Subject Category | |
| --- | --- |
|  | Subjects admitted strictly for research purposes. Hospitalization and laboratory costs are paid by the funding source. |
|  | Research subjects receiving established medical care. Hospitalization and laboratory costs paid by third party. (except for tests performed exclusively for the study) |
|  | Research subjects admitted on an industry-sponsored protocol. All costs paid by industry sponsor. Requires $2,200 IRB application and set-up fee. |

| ClinicalTrials.Gov Registration | |
| --- | --- |
| Does this study need to be registered on [www.clinicaltrials.gov](http://www.clinicaltrials.gov/)? (see below) | No  Yes |
| General Requirements [U.S. Public Law 110-85](http://frwebgate.access.gpo.gov/cgi-bin/getdoc.cgi?dbname=110_cong_public_laws&docid=f:publ085.110.pdf) (Food and Drug Administration Amendments Act of 2007 or FDAAA), Title VIII, Section 801 mandates that a "responsible party" (i.e., the sponsor or designated principal investigator) register and report results of certain "applicable clinical trials":   - **Trials of Drugs and Biologics:** Controlled, clinical investigations, other than Phase I investigations, of a product subject to FDA regulation; - **Trials of Devices:** Controlled trials with health outcomes of a product subject to FDA regulation (other than small feasibility studies) and pediatric post-market surveillance studies.   "Applicable clinical trials" generally include interventional studies (with one or more arms) of drugs, biological products, or devices that are subject to FDA regulation, meaning that the trial has one or more sites in the U.S, involves a drug, biologic, or device that is manufactured in the US (or its territories), or is conducted under an investigational new drug application (IND). | |

| Investigator’s Assurance |
| --- |
| The Principal Investigator must assure the IRB that all procedures performed under the project will be conducted in strict accordance with all applicable federal, state and local regulations and laws regarding the protection of human subjects in research including, but not limited to:  - Use of qualified personnel to conduct the project according to the protocol approved by the IRB. - Ensuring that no changes are made to the approved protocol or consent form without prior IRB approval (except in an emergency to safeguard the well-being of subjects). - Using the most current, approved, IRB stamped consent form to obtain informed consent from subjects or their legally responsible representative. - Prompt reporting to the IRB in writing of any changes in research activity, unanticipated problems involving risks to subjects or others, and adverse events (AEs), within the time period specified by IRB policy. - If I will be unavailable to direct this research personally, as when on leave or vacation, I will arrange for a co-investigator to assume direct responsibility in my absence. If this is not a co-investigator named in my absence, I will notify the IRB in writing of the responsible party.   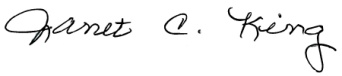  8/17/10  Principal Investigator’s Signature Date |

| Faculty Sponsor’s Assurance |
| --- |
| By my signature as sponsor on the research application, I certify that the student/investigator listed on page one is knowledgeable about the regulations and policies governing research with human subjects and has sufficient training and experience to conduct this particular study in accord with the approved protocol. In addition,   - I agree to meet with the student/investigator on a regular basis to monitor study progress. - Should problems arise in the course of the study, I agree to be available to personally supervise the student in solving them. - I assure that the student/investigator will promptly report Adverse Events to the IRB according to the schedule indicated above. - If I will be unavailable, as on vacation, I will arrange an alternate faculty sponsor to assume responsibility during my absence and I will advise the IRB of such arrangements.     Signature of Faculty Sponsor Date  (if co-investigator is a student, resident, or fellow) |

| Statement of Financial Interests |
| --- |
| By the signatures below, each investigator is certifying that either no financial interest exists or a complete listing of all financial interests related to the proposed project is provided. All individuals named below further acknowledge their responsibility to disclose any new reportable financial interest obtained during the term of the project. The Principal Investigator’s signature also certifies that all individuals required to make disclosures have been listed below: (Attach additional sheet if necessary) |
| Do you, your spouse, or dependent children, have a financial interest in the work to be conducted under the proposed project?  **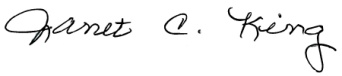**  8/17/10  No  Yes: Attach Financial Disclosure Form  Signature of Principal Investigator Date  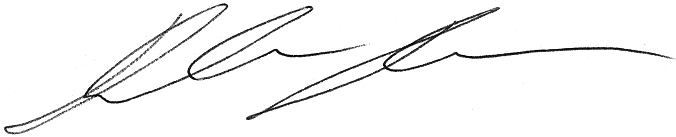  8/20/10  No  Yes: Attach Financial Disclosure Form  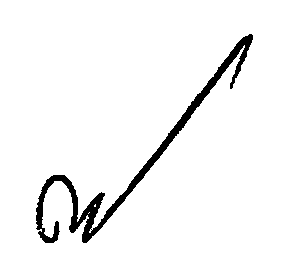 Signature of Principal Investigator Date  8/20/10  No  Yes: Attach Financial Disclosure Form  Signature of Principal Investigator Date  No  Yes: Attach Financial Disclosure Form  Signature of Co- Investigator Date  No  Yes: Attach Financial Disclosure Form  Signature of Co- Investigator Date |

| Documentation of Investigator Education in Human Subject Research |
| --- |
| Training in Human Research Subject Protections is required for all individuals who are participating in research activities at Children’s Hospital & Research Center Oakland and Children’s Hospital Oakland Research Institute. The Principal Investigator, Co-Investigators and other study staff interacting with research subjects must complete the University of Miami School of Medicine CITI Program in the Protection of Human Research Subjects to obtain IRB approval of a new study. Core Modules (Basic Course) must be completed only once, and Continuing Education Modules (Refresher Course) must be completed annually. (Attach additional sheet if necessary for other study personnel.) |
| Janet King, Ph.D. R.D.  Basic/Refresher CITI Course Completed  Name of Principal Investigator  Deborah Dean, M.D., M.P.H.  Basic/Refresher CITI Course Completed  Name of Principal Investigator  Tu Ngu, M.D., Ph.D.  Basic/Refresher CITI Course Completed  Name of Principal Investigator  Basic/Refresher CITI Course Completed  Name of Co-Investigator |

| **Summary Information** | | | | | | | | | | |
| --- | --- | --- | --- | --- | --- | --- | --- | --- | --- | --- |
| **Age Range of Eligible Subjects:** 18 to 30 years | | | | | | | | | | |
| **Subject Population:** (Please check all that apply) | | | | | | | | | | |
|  | a. neonates | |  | f. minors | | | | |  | k. cancer patients |
|  | b. minorities/immigrants | |  | g. normal volunteers | | | | |  | l. terminally ill |
|  | c. comatose patients | |  | h. institutionalized | | | | |  | m. students |
|  | d. decisionally impaired | |  | i. wards of the court | | | | |  | n. pregnant women |
|  | e. elderly | |  | j. prisoners or parolees | | | | |  | o. |
| **Study Type:**  If the research involves any of the following, please check all that apply. | | | | | | | | | | |
|  | | a. Investigational Drug (IND) | | | |  | | l. Investigational Device (IDE – HUD) | | |
|  | | b. Genetic Research (DNA) | | | |  | | m. Vaccine Trial | | |
|  | | c. Collection of Biological Specimens for Banking and/or Collection of  PHI (identified data) for a Database | | | |  | | n. Transplantation | | |
|  | | d. Collection of Remnant Surgical Specimens | | | |  | | o. PI or Co-PI is the treating clinician | | |
|  | | e. Magnetic Resonance Imaging (MRI) | | | |  | | p. Radiation (including X-ray, DXA) | | |
|  | | f. Gene Transfer Therapy | | | |  | | q. Biohazardous Waste | | |
|  | | g. HIV Screening | | | |  | | r. HIV/AIDS Research | | |
|  | | h. Alcohol and Drug Abuse Research | | | |  | | s. Controlled Substances | | |
|  | | i. Acute Care Waiver of Informed Consent | | | |  | | t. Deception | | |
|  | j. Behavioral Observations | | | |  | | u. Audio/Videotapes or Focus Groups | | | |
|  | | k. Surveys, Questionnaires or Psychological Testing | | | | | | | | |
| **Data Safety Monitoring: *All interventional studies involving greater than minimal risk must include a Data Safety Monitoring Plan (DSMP).*** A DSMP is a plan established to assure that each research study has a system for appropriate oversight and monitoring of the conduct of the study to ensure the safety of participants and the validity and integrity of the data. The DSMP should indicate specifically whether or not there will be a formal Data Safety Monitoring Board (DSMB) or Data Monitoring Committee (DMC). | | | | | | | | | | |

| Has a Data Safety Monitoring Board been established to review data and/or adverse events related to this study? | | | | | N/A (minimal risk)  Yes  No | | | |
| --- | --- | --- | --- | --- | --- | --- | --- | --- |
| Describe the DSMB: | | | | | | | | |
| The DSMB is composed of professionals from CHORI and the National Institute of Nutrition in Hanoi. The Board will meet at least twice/year via conference call. Additional meetings will be arranged as necessary.    David Durand, M.D.  Priscilla Joe, M.D.  Frank Wierenga, M.D., Ph.D. (IRD office at the NIN) | | | | | | | | |
| **Research Sites:** Except for multi-center clinical trials (e.g., industry, COG) list all sites in which the research is to be conducted and attach other IRB approval letters. If applicable, attach letters of support from those institutions.  N/A – Multi-center clinical trial | | | | | | | | |
| National Institute of Nutrition, Hanoi, Vietnam | | | | | | | | |
| **Investigational drugs/devices:** If any investigational drugs or biologic agents are used in this study, please include three copies of the Investigational Drug Information or Investigator’s Brochure (IB) with this application.  **N/A IB Number:       Version Date of IB:**  **Complete and attach the following IRB forms (on the website) as applicable:**  **Study Review - Investigational Drug Information**  **Study Review - Investigational Device Information** | | | | | | | | |
| Protocol Summary | | | | | | | | |
| **Protocol Version Date: 08/17/10 Amendment #:**  **Protocol Number: Version 1**  **N/A (no number)** | | | | | | | | |
| Please complete the requested information in the categories below. If the item does not apply to your research, please indicate that the question is not applicable. The information should be intelligible to IRB reviewers from a variety of lay and scientific backgrounds. | | | | | | | | |
| **Hypothesis:** Briefly explain the hypothesis(es) to be tested. If the study is not designed to test a hypothesis, simply state “None.” | | | | | | | | |
| We hypothesize that ASF supplementation from pre-conception to term (PC-T) improves maternal health [increased nutrient status, decreased infections, increased gestational T helper 1 (Th1) responses], which in turn contributes to significantly increased fetal growth, increased birth weight and decreased prematurity compared with ASF supplementation from mid-gestation to term (MG-T) or routine prenatal care. | | | | | | | | |
| **Purpose of the study:** What are the specific scientific aims of this study? | | | | | | | | |
| Aim 1: To evaluate the effects of ASF supplementation from PC-T or MG-T compared with routine prenatal care on maternal nutrient status (iron, zinc, vitamin A, and B_12_ status);  Aim 2: To evaluate the effects of ASF supplementation from PC-T or MG-T compared with routine prenatal care on incidence of infections, immune and stress responses, insulin resistance, and placental weight;  Aim 3: To compare the effects of ASF supplementation from PC-T or MG-T compared with routine prenatal care on the outcomes of fetal growth, birth weight, the prevalence of pre-term births, and infant growth during the first six months of life; and correlate data in Aims 1 and 2 with the birth outcomes. | | | | | | | | |
| **Background and Significance:** Include a brief summary of previous work that provides a basis for the proposed research and that supports the expectations of obtaining useful information without undue risk to human subjects. **Provide a Bibliography (References)**  This information aids IRB reviewers in assessing how valuable the project is likely to be. If graphs or tables are used to convey information, please maintain a consistent style and make sure that fonts are no less than 11-point in size. If no preliminary data are available, it may be helpful to briefly indicate why this proposed study is a reasonable starting point. Note that some IRB members are non-scientists and may not be familiar with scientific or technical terms. | | | | | | | | |
| It has been known for over 80 years that maternal starvation reduces fetal growth and increases neonatal infections (1). Consequently, programs have been initiated to improve the fetal nutrient supply by providing food and/or micronutrient supplements to women after they become pregnant. The impact of these efforts has been disappointing. In developing countries, the prevalence of low birth weight (LBW) and infant mortality remain high; ~16% of newborns are LBW and the infant mortality rate is >50/1000 live births (2). More recently, as a result of improved access to cereals following the ‘green revolution,’ nutritional concerns shifted from protein and energy to micronutrient deficiencies, especially those in animal-source foods (ASFs)—iron, zinc, vitamins A and B_12_. Thus, a UNICEF/UNU/WHO working group recommended in 1999 that pregnant women in developing countries be given multiple micronutrient (MMN) supplements rather than only iron and folate (3).  Results of MMN supplementation programs have also been disappointing for birth weight; a meta-analysis of 13 MMN studies showed only a 54 g increase in birth weight (4). Most of those studies were initiated in mid- to late pregnancy when energy demand for fetal growth is high and too late for MMNs to enhance maternal metabolic processes that improve fetal nutrient transfer. As animal studies suggest, initiating MMN supplements prior to or during early pregnancy when oocyte and embryo development, placental function, and maternal metabolic adjustments are established may be more effective than gestational interventions (5). Our study of inter-pregnancy (between pregnancies) food supplementation showed that supplementation for 5 to 7 months before the second pregnancy increased birth weight significantly more than supplementation for <2 months (6). Insufficient intakes of micronutrients also increases the risk for maternal morbidity (7). Increased rates of maternal infection may enhance the prevalence of LBW by increasing prematurity rates (2). Vitamin A, zinc, and iron all play essential roles in maintaining a coordinated immune response (8). Thus, low intakes of ASFs prior to conception may increase maternal infections during gestation, increase pre-term births, and increase delivery of LBW babies.  Our goal, therefore, is to compare the effects of an ASF supplement given from pre-conception to term (PC-T) or from mid-gestation to term (MG-T) with routine prenatal care on maternal nutrient status; incidence of infection; the immune and stress responses; and fetal growth, infant birth weight and prematurity, and infant growth during the first 6 months of life. The study will be performed in rural Vietnam where ~40% of women are anemic, ~50% have infections, and ~10% deliver LBW babies (9). Rice is the diet staple, and ASF consumption is rare. Our Preliminary Studies show that not only is an ASF supplement program feasible and sustainable in rural Vietnam but the proportion of women with inadequate intakes of iron, zinc, vitamin A, and vitamin B_12_ intakes range from 24 to 83%. Since folic acid intake is also low, we will add dark green vegetables, a rich source of folic acid, to the ASF supplement.  Currently, international nutrition programs for women are not initiated until pregnancy. Given the disappointing impact of these programs, we propose a major paradigm shift by performing an innovative study of a micronutrient-rich ASF supplement, which is readily available and sustainable long-term in rural Vietnam, given to women from PC-T compared head-to-head with ASF supplementation from MG-T or with routine prenatal care. This will be the first study to not only assess maternal MMN status before conception but also to correlate MMN status with maternal immunity, stress and infection throughout pregnancy, and the effects of these on fetal growth, birth weight and prematurity. This comprehensive approach will advance new knowledge about the interaction of nutrition and immune status that could substantially influence the field of nutrition. If the ASF or a local foods approach that provides limiting micronutrients for PC-T improves pregnancy outcomes more than a gestational intervention, this will change the concepts, treatment, and preventive interventions for prenatal nutrition worldwide. | | | | | | | | |
| **Study Design:** (Check all that apply). | | | | | | | | |
| Placebo | Blinded | Randomized | Investigational intervention without random assignment | | | | | |
| If this study has any of the formal designations below, please indicate below:  N/A | | | | | | | | |
| Phase I | Phase II | Phase III | Phase IV | Open Label Extension | | | | |
| Additional description of general study design. Sequentially list all procedures, drugs or devices to be used on human subjects. Describe any use of placebos and indicate whether subjects will be randomized in this study. Attach flow diagram if appropriate. **If there are any investigational drugs, devices or biologic agents used in this study, please complete and attach the FDA Form 1572.** If this is an investigator-initiated study, attach the FDA Investigational Drug Application. | | | | | | | | |
| The study is an unmasked, ~~cluster,~~ randomized trial. A total of 1044 women from 174 villages in 14 rural communes will be recruited when they register to marry. The women will then be randomly assigned ~~by village~~ to one of three interventions: I) ASF supplement (~150 kcal, ~14 mg iron, 5.2 mg zinc, 1100 µg RAE vitamin A, 9 µg vitamin B_12_, and ~500 μg folic acid) 5 days/wk from marriage to term (~13 months); II) ASF supplement 5 days/wk from 16 wks gestation to term (~5 months); or III) routine prenatal care. Nonanemic women will not be given iron and folate supplements during pregnancy. However, if a woman’s hemoglobin is below 11 g/dL at any time during the study, she will be given 60 mg iron and 400μg folate, as recommended by the WHO. Her hemoglobin will be tested again after 2 months. If it is remains low, iron and folate supplementation will continue for another 2 months. Re-testing every 2 months will continue until the hemoglobin is greater than 11 g/dL. The primary outcome will be birth weight and the secondary outcome will be the prevalence of prematurity. Other outcomes include infant growth during the first 6 months of life, maternal micronutrient status and anemia, the incidence of infections (urinary tract infection, trachoma, pneumonia, and diarrhea); infant growth and infections (pneumonia and diarrhea) from 0-6 months of age will also be assessed. Maternal dietary intakes, height, weight, mid-upper arm circumference, triceps and subscapular skinfold thickness; iron, zinc, vitamin A and B_12_ , and folate status, and immune function will be measured at recruitment, 16, and 32 weeks gestation. Incidence of infections will be assessed twice-monthly from enrollment through six months postpartum by a standardized questionnaire administered by a health worker for all three groups along with rapid urine assessment. We will also assess hours per day worked in the field. Infant weight, length, head, mid-arm, and abdominal circumference will be measured at birth, 2, 4, and 6 months. Infant gestational age will be assessed at birth to determine the prevalence of pre-term deliveries. Maternal and infant medical records will be reviewed at the end of the study to obtain information regarding the incidence and treatment of medical problems. | | | | | | | | |
| Study Population | | | | | | | | |
| **Characteristics of the Subject Population:**  Describe the gender, ethnic background and health status. Provide a candid discussion of potential problems, if any, related to the study population. Explain the rationale for the use of special classes such as pregnant women, children, prisoners, wards of the court, or other vulnerable populations. If women, minorities or children are excluded, provide written justification. | | | | | | | | |
| Our subject population includes nulliparous women between 18 and 30 years old registering to marry in the Cam Khe District of Phu Tho Province in rural northern Vietnam and planning to have children. These women are predominantly (>99.9%) Kinh ethnicity, and about 95% are farmers as their primary occupation. Young couples in Cam Khe register for marriage one to two months before marriage. In 2006 and 2007, more than 95% of the couples who registered began their first pregnancy within one year after marriage. About 50% of deliveries are at the district hospital, which is 15 km (a 45 minute drive in good weather) from the furthest commune in the district. The remaining 50% of deliveries are at the commune health stations. Currently, home births are less than 1% of all births, whereas 15 years ago nearly half of all births in the district were at home.  The primary health problems these women face in pregnancy are low birth weight (LBW) and prematurity. Although the prevalence of LBW has declined significantly in urban areas of Vietnam, it still is unacceptably high among rural women living in mountainous, farming regions. For example, the prevalence of LBW declined from 8.2 to 4.3% in males and from 10.7 to 6% in females between 1981-84 and 1997-98 in Hanoi (10). In 1996, LBW incidence in rural women in northern Vietnam ranged from 7.9 to 12.5% (11). Farming mothers are twice as likely to have a LBW delivery and three times as likely if their BMI was under 18.5. Most of the farming mothers subsist on rice and vegetables only and about 94% have an insufficient food intake as assessed by energy consumption. It is unknown if the higher rates of LBW among rural women are due to intra-uterine growth retardation or pre-term deliveries. The rate of pre-term deliveries in Hanoi is about 11.8% and, although assessments of gestational age are inadequate in rural areas, it is thought that the rural pre-term delivery rates are as high as 36% among women doing farm work (12). | | | | | | | | |
| **Inclusion/Exclusion Criteria:** Indicate the criteria for exclusion and inclusion and explain the system for equitable selection of subjects. | | | | | | | | |
| **Inclusions**: Non-pregnant women registering to marry in the Cam Khe District of Phu Tho Province, nulliparous, between 18-30 years of age, and planning to reside in the study site for the next 3 years.  **Exclusions**: Women who smoke, are already married, have had a previous pregnancy, have a history of severe infection (i.e., HIV, tuberculosis, or malaria) or a chronic disease (i.e., diabetes, cardiovascular or renal disease, or severe energy deficiency with BMI < 17 kg/m^2^), do not reside in the study communes, or are unable to provide informed consent. | | | | | | | | |
| 1. **Eligibility for Study:** How is eligibility determined and by whom? | | | | | | | | |
| A screening questionnaire listing in detail the exclusion and inclusion criteria will be used at recruitment. Study staff will discuss the criteria with the potential subject and complete the questionnaire. If the subject is negative for any of the inclusion criteria then they will be thanked for their time and dismissed from further study activities. The principle investigator and/or study coordinator will review each subject's screening questionnaire, approving them for participation in the study. | | | | | | | | |
| 1. **Duration of Subjects' Participation in the Study (include follow-up if applicable):** | | | | | | | | |
| Recruitment (within 1 month before marriage) until 6 months postpartum. | | | | | | | | |
| Enrollment Plan and Recruitment | | | | | | | | |
| 1. **Planned Enrollment at CHRCO:**  Number of subjects needed to complete the study. | | | | | | | | 1044 |
| **Enrollment plan:** If you expect failed screenings or subject withdrawals, will they be replaced until the appropriate numbers of subjects have completed the study? If No, explain below. | | | | Yes  No | | | | |
| N/A | | | | | | | | |
| **Recruitment:** What methods will be used to identify and recruit potential subjects? Attach a copy of all planned advertisements, flyers and letters, etc. to potential subjects. | | | | | | | | |
| Subjects will be recruited through marriage registration logs for the Cam Khe District, Phu Tho Province, and through additional advertising by health station personnel and the local Women’s Union in each of the participating communes. Women between 18 and 30 years old who have registered to marry and who plan to become pregnant will be invited to the health station for a screening appointment. If the woman meets our study criteria, she will be invited to participate in the full study. If the rate of recruitment is slower than anticipated, we will extend the recruitment to additional sites in the Cam Khe District. | | | | | | | | |
| Research Methods and Procedures | | | | | | | | |
| 1. **Methodology and Data Collection:** Describe the research procedures that will be followed. Please list, in sequence, all study procedures, tests, and treatments required for the study. Please indicate those that are experimental and those that may be considered to be standard treatment. Include a detailed explanation of any experimental procedures. Attach table if available. Describe all activities involving human subjects and explain the frequency and duration of each activity. | | | | | | | | |
| *A table is attached to provide a summary of procedures performed at each study visit.*  Screening visit: Subjects will review and sign the consent form and complete a detailed health history questionnaire. Menstrual pattern, previous illnesses, maternal height, weight, mid-upper arm circumference, triceps and subscapular skinfold thickness will be measured using standardized procedures (13). Health workers will be trained in anthropometric methods; all measurements will be made in duplicate. A 24-hour dietary recall will be collected at this time. This visit will last approximately 1 hour.  *Maternal Anthropometry:* Maternal height will be measured in the standing position using a non-stretchable tape and a right-angled headboard attached to a wall. Standing height will be measured without shoes. Maternal weight will be measured at the screening visit, 16 and 32 weeks gestation and 2, 4 and 6 months postpartum using a high-quality electronic scale to the nearest 0.1 kg. The women will be measured in light underclothing and paper gowns. The scales will be calibrated regularly with a set of standard weights. The body mass index (BMI) will be calculated from the weight (kg) ÷ height (m)^2^. Women with BMI <17.0 at recruitment will be excluded from the study. Maternal triceps and subscapular skinfold thickness will be measured using calibrated Lange calipers on the non-dominant side. The mid-upper-arm circumference will be measured at the same site as the triceps skinfold thickness. The mid-upper-arm fat area, determined from measurements of mid-upper-arm circumference (MUAC) (mm) and triceps skinfold thickness (TSK) (mm), equals (TSK x MUAC/2) – (π x TSK^2^ ÷ 4) (13). Mid-upper-arm muscle area (MUAM), determined as an estimate of fat-free mass, will also be calculated: MUAM= [(MUAC – (π x TSK^2^)/4π] – 6.5 (14). The dietitians responsible for doing the dietary recalls will be trained in the anthropometric methods; all measurements will be made in duplicate.  Visit 1: Baseline (t_0_): At their respective commune health station, subjects will have their blood pressure measured. A fasting blood sample will be taken for measurements of hematological parameters, micronutrients, and Th1/Th2 cytokines as detailed below. Since the hydration status of woman at the time of blood draw influences the concentration of blood components, we will give the women a 12-oz bottle of water to drink upon rising the morning of the blood draw. In our preliminary studies, we found the women sometimes forget and consume food prior to the blood draw. The nurses will question all women about food intake before drawing the blood. If they have done so, the blood draw will be rescheduled within a week. The participant will also provide a spot urine sample.  *Fasting blood samples:* [25 mL total: 2 x 5 mL in heparinized tubes; 2 x 5 mL in serum separator tubes (5 mL aliquot for serum micronutrient assays and 5 mL aliquot for cytokine/chemokine assays) and 1 x 5 mL into EDTA tubes (1 mL for hematology and 4 mL for PBMC separation for future studies)] will be collected at Commune Health Stations at baseline, at 16, and 32 weeks gestation. Samples will be centrifuged in the field within one hour of collection at 2500 × *g* for 15 minutes; plasma/serum samples will be separated into aliquots and placed on ice until transported to -20°C study freezers located at the District Hospital. To avoid degradation of vitamin A by light exposure, all samples will be collected and processed indoors. At the end of each week, all samples will be transported to the NIN in Hanoi and stored at -80ºC.  *Iron status and supplementation:* Iron status be assessed from measurements of hemoglobin, red blood cell number, hematocrit, mean corpuscular volume, red blood cell distribution width using a Celltac-α semiautomatic analyzer (Nihon Kohden, Tokyo, Japan), plasma ferritin and transferrin receptor (TfR) using ELISA methods (Ramco Laboratories, Stafford, TX). Anemia will be identified using Institute of Medicine standards for pregnancy (15). Iron deficiency will be defined as a TfR level > 8.5 mg/L and a plasma ferritin < 20 mg/L. Iron deficiency anemia (IDA) will be defined as a low Hb and iron deficiency. Whole body iron will be estimated from the ratio of TfR and ferritin (16). If a woman is diagnosed with IDA at any time during the study, she will be given 60 mg supplemental iron daily for two months prior to evaluating her hemoglobin again. If she is still anemic, she will receive 60 mg supplemental iron/d and 400 μg folate/d for another two months; if not, supplemental iron therapy will be discontinued.  *Zinc:* Plasma samples will be thawed and wet-digested using trace element free nitric acid in acid-washed tubes, and zinc concentrations will be determined by flame atomic absorbance spectrometry as previously described (19, 20). Values < 10.7 µmol/L in non-pregnant women and < 8.6 µmol/L in pregnant women will be considered deficient (21). Women with low values will be noted, but they will continue in the study. Serum alkaline phosphatase activity will also be measured as a secondary measure of zinc status.  *Vitamin A:* Plasma samples will be thawed and treated with ethanol to denature proteins, then retinol will be extracted twice with hexane under gold fluorescent lighting to minimize degradation of retinol. Purified retinyl acetate dissolved in ethanol will be used as an internal control to assess the efficiency of extraction. Hexane layers will be pooled and evaporated under nitrogen. The residues will then be reconstituted to a mixture of methanol and tetrahydrofuran. Extracted retinol will be determined by HPLC at 320 nm (22). Serum retinol binding protein (RBP) will be determined by ELISA. A serum vitamin A level below 0.70 µmol/L and a serum RBP below 0.70µmol/L will be considered deficient (13).  *Vitamin B_12_:* Concentrations of B_12_ as free cobalamin in serum will be determined using a commercially available fluorometric competitive binding assay (PerkinElmer, Waltham, Massachusetts). Serum values < 130 pmol/L will be considered below normal in our subjects (13).  *Folic Acid:* Concentrations of folate in serum and erythrocytes will be determined using high performance liquid chromatography (HPLC). Serum values <6.8 nmol/L or erythrocyte values <363 nmol/L will be considered below normal in our women (13).  *Study Questionnaire:* Maternal morbidity will be assessed at baseline and twice-monthly from enrollment to 6 weeks postpartum using the Study Questionnaire (see Appendix) administered by the Health Station doctors. We will determine the presence of 1) a UTI from signs and symptoms including urgency, frequency of urination, burning, pain, blood in the urine, or foul-smelling urine; 2) presumptive pneumonia from signs and symptoms including sputum production or dry cough with or without fever [>39°C (102.2°F)]; and 3) diarrhea (defined as loose, watery stools more than three times a day for >2 days) with or without fever, blood or mucous in the stool or vomiting. The Study Questionnaire will also determine the number of hours of physical work each day, type of work, and whether the mother feels quickening or false labor contractions when she bends at the waist during her work. These data will be used to determine any interaction between maternal physical work and ASF supplementation on birth weight and preterm delivery.  *Urine sample and dipstick parameters:* Urine will be collected using the midstream technique after the patient is instructed to clean the labia three times with three different sterile wipes provided to her. An aliquot of urine will be used immediately for dipstick (Bayer Multistik) analysis of basic urinary chemistry, including pH, protein, glucose, ketones, nitrites, leukocyte esterase, ascorbic acid, bilirubin, and presence of blood using the Urine Dip Stick strips. Two 2-mL aliquots will be saved for future studies. A leukocyte esterase level > 1 and a positive reading for nitrites will be used to diagnose a UTI; presence of trace blood or greater value on the dipstick may indicate hemorrhagic cystitis. In this case, the woman will be referred to the District Hospital for further evaluation and treatment; In addition, all pregnant study participants will be tested for a UTI regardless of symptoms [because the majority of women during pregnancy will have asymptomatic UTIs (23)] using a urine dipstick at the Health Station at 16, 24, and 32 weeks gestation. A study by Lammers et al. found that urine dipsticks were as accurate as urinalysis in diagnosing a UTI (24).  *Eyelid and tear samples*: Eye drops will be put into the eyes to numb the eyelid. The upper eyelid will be flipped and gently swabbed. This will be done carefully by an experienced and qualified staff ophthalmologist or trained field person. Tears will also be collected by placing an ocular sponge in the inner canthus and allowing the sponge to become saturated with the tears. The tear samples will be analyzed for presence of Chlamydia trachomatis.  *Dietary recalls*: A 24-hour dietary recall will be collected at the screening appointment and a repeat baseline 24-hour dietary recall will be collected at the subject’s home within the week before the baseline sample collection. Additional 24-hour dietary recalls will be collected twice each at 4 weeks after enrollment, at 16 and 32 weeks gestation (8 recalls total per subject). Measurements are made on two different days to assess *usual* intakes as previously described (25, 26). Our dietitians are trained in the multi-pass dietary recall method using Vietnamese food models and typical eating patterns (22). Vietnamese food composition tables will be used to evaluate energy, protein, iron, zinc, vitamin A, vitamin B_2_, vitamin B_6_, folic acid, and vitamin B_12_ intakes (27, 28). This comprehensive approach toward nutrient intakes will allow us to evaluate the impact of supplemental ASF on usual intakes and the relationship of total diet (usual diet plus supplement) on our outcome measures.  *Study Supplements:* A 10-day rotating cycle of local ASF supplements will be prepared by highly-trained cooks and provided to women in the intervention groups PC-T (beginning after the baseline sample collection) and MG-T (beginning at 16 weeks gestation) for 5 days/week. Ingredients will be bought fresh early each morning at local markets by the five cooks who will prepare the supplement at 5 of the 20 Commune Health Stations that are centrally located. After ASF supplements are prepared and portioned into servings for each participant, 20 ASF workers will distribute the supplements to the women at mid-morning either at the village or Commune Health Station by motorbike. The supplement will always be eaten outside the home to avoid the influence of customary household food distribution priorities. Consumption of the supplement will be monitored by the same ASF workers and the amount consumed per woman will be recorded in the Daily ASF Intake Log that the ASF workers will maintain and show to Dr. Hall and Ms. Hong Anh weekly. Dr. Hall and the two study dietitians from NIN will train the cooks and do frequent, random checks on ASF preparation. Samples of the ASF supplement will be collected randomly from each of the kitchens twice monthly throughout the study and analyzed at NIN for the four controlled nutrients (iron, zinc, vitamins A and B_12_ and folate) to assure consistency within and among the kitchens. Drs. Ngu and Hall will randomly check supplement ingredients and weights in the kitchens weekly to assure that quality is maintained. The cooks will be instructed in food safety and hygiene. Fresh ingredients purchased daily will be prepared under sanitary conditions within one hour of purchase. The ASF supplements will be stored in heat-insulated containers from the time they are cooked until consumed in the field, which will be <2 hours. All uneaten food will be disposed of immediately following distribution to the women. During our pilot study of 117 women, using these hygiene and food preparation protocols in three communes over six months, all food was consumed with no incidents of food poisoning.  *Evaluation of Compliance:* Each ASF Worker will have a Daily ASF Intake Log of all women eligible to receive ASF in the villages for whom they are responsible. The ASF worker will record whether the woman came for her supplement or not, the amount consumed (1/4, 1/2, 3/4ths or all), reason for not consuming the entire supplement, and any comments or complaints from the woman. These logs will be reviewed with Dr. Ngu or Dr. Hall on a weekly basis. Any participant missing >10 supplements in a two-month period, except in the case of illness (captured in Study Questionnaire, see Appendix), will be considered non-compliant and dropped from the study (i.e., dropping below 75% compliance over a 2-month period). We have assumed a 15% attrition rate due to non-compliance or other reasons. In our pilot study, overall compliance was 98.5% during the 6-month intervention.  Visits 2, 16 weeks gestation (t_1_): Blood pressure, uterine height, anthropometry, urine sample, eyelid swab and tears, first ultrasound biometry.  *Ultrasound biometry:* The incidence of pre-term births is our secondary outcome. The gestational age of the baby will be estimated by ultrasound at the 16 week measurement. We will advise all women to mark the first day of their last menstrual period on a small calendar given to them when they are enrolled in the study and to report to the Health Station for an evaluation within 4 weeks of missing a period. We will use the first day of the woman’s last menstrual period to confirm gestational age estimated from the ultrasound exam. A pre-term birth will be defined as occurring before 37 completed weeks of gestation, a very pre-term birth before 34 completed weeks of gestation, and a severely pre-term birth occurring before 28 weeks gestation. We will determine the biparietal diameter, head circumference, abdominal circumference and femur diaphysis length. Reference data have been established for each of these parameters, and fetal weight can be calculated. Since choice of reference data can lead to up to a 4-fold increase in determinations of fetal abnormalities (29), we will calculate Z-scores (30) from local ultrasonography data to determine the most appropriate reference data for our study population (29). The most sensitive indicator of symmetric and asymmetric intrauterine growth retardation (IUGR) is the abdominal circumference, which has a sensitivity of over 95 percent if the measurement is below the 2.5th percentile (31, 32). Accurate dating of the pregnancy is essential in using any parameter. We will also use the ratio of the head circumference to the abdominal circumference (HC/AC) to identify asymmetric growth restriction (33).  Visit 3, 24 weeks gestation: Blood pressure, uterine height, anthropometry, urine sample, second ultrasound biometry.  Visit 4, 32 weeks gestation (t_2_): Blood pressure, uterine height, anthropometry, maternal blood and urine samples, third ultrasound biometry, eyelid swab and tear samples.  Visit 5, Birth: Infants are routinely delivered in the Health Station or at the District Hospital. Therefore, we will train our delivery room nurses to precisely measure birth weight using standardized balances. Infant length and head circumference will also be measured at birth. Naked weights will be made using a Seca 334 infant scale accurate to 10 g (Seca, Corp, Hanover, MD) available at each Health Station. Recumbent length will be measured using a wooden measuring board by two trained nurses. One will apply gentle traction to bring the crown of the child’s head into contact with the fixed headboard; the second will hold the baby’s feet, without shoes and with toes pointing directly upward, and keeping the knees straight, bring the movable footboard to rest firmly against the heels. Readings will be made to the nearest 0.1 cm (13). The head circumference will be measured at the same time points using a narrow, flexible, non-stretch tape; the measurement is made at just above the supraorbital ridges over the most prominent part of the frontal bulge and the occiput giving the maximum circumference. Mid-arm circumference will also be measured. The abdominal circumference will be measured over the umbilicus using the flexible tape measure to assess infant fatness (34). Infant triceps skinfold thickness will be measured using calibrated Lange calipers. Duplicate measurements will be made of each parameter. The infant anthropometric measurements will be repeated at 2, 4, and 6 months of age at clinic visits by trained nurses.  *Placenta weight:* Delivery room nurses will weigh (using the same scale after the newborn is weighted) the placenta according to a standardized procedure that specifies length of time for cord clamping and cleaning the placenta surface with absorbent towels before weighing on an electronic balance [95]. Except for rare occurrences when women are unable to get to the Health Station because of a washed-out road or short labor, deliveries routinely take place in delivery rooms. Since trained study nurses will attend all study women, we do not anticipate significant problems with obtaining placenta weights. In the event of a home delivery, all participants will be instructed to keep the placenta for weighing by the nurses  Visit 6, Infant follow-up, 2 months: Infant anthropometry; maternal urine, eyelid swab and tears.  Visit 7, Infant follow-up, 4 months: Infant anthropometry.  Visit 8, Infant follow-up, 6 months: Infant anthropometry, Bayley scale, maternal urine, eyelid swab and tears.  *Bayley Scale:* To assess infant developmental outcomes, a Bayley Scale validated for use in rural Vietnam (National Hospital of Pediatrics, Hanoi, Vietnam) will be conducted on each infant twice by rural doctors: two weeks before 6-months age, and at 6-months age. For quality control, a pediatrician from Hanoi will conduct a third (triplicate) scale at random in 10% of the infants assessed by each of the local doctors. | | | | | | | | |
| 1. **Surveys, questionnaires, or psychological tests:**  If applicable, please describe the provisions for administering these measures, the mode of administration, the setting, and if special training or qualifications are necessary.  N/A | | | | | | | | |
| *Screening questionnaire:* Pham Hong Anh or Hoang Thu Nga will administer the screening questionnaire at the women’s homes. This questionnaire will determine eligibility for the study, and will also be used to collect information about socioeconomic status, occupation, education, hygiene, family members, use of nutritional supplements, alcohol, and medications, and history of diseases and infections.  *Study questionnaire:* The Health Center Doctors will assess the incidence of infections in all of the women using a standardized questionnaire (see Appendix) every two weeks from 16 weeks gestation to 6 months postpartum. The two monthly assessments will alternate between the Health Center and the woman’s home. Questions regarding the incidence of urgent, frequent urination with burning or pain, blood in the urine, or foul-smelling urine will be asked to assess a potential urinary tract infection (UTI); the incidence of a productive cough with or without fever and or diarrhea with or without vomiting during the past week will also be determined. If the conditions warrant treatment, the Doctors will provide advice and prescribe routine treatment. If necessary, the Doctor may recommend further care at the District Hospital. After the infant is born, appropriate questions will be asked about the incidence of infections (diarrhea and pneumonia) in the newborn at the same time as when the mother is evaluated. This questionnaire will also determine the number of hours of physical work each day, the type of work done, and whether the mother feels quickening, or false labor contractions, when she bends at the waist during her work.  *24-hr dietary recalls:* Dieticians trained in quantitative dietary recall interviews by NIN staff will administer the 24-hr recalls. The interviews will be conducted at the women’s homes, and involve a detailed interview of foods consumed and portions used in the previous 24-hour period. The dietician will use a spring balance and standard measures of volume to estimate quantities of foods consumed based on the subject’s recollection and based on dishes, utensils, and (when possible) leftover foods from the subject’s home. A 24-hour dietary recall interview will be conducted at the screening appointment and a repeat baseline 24-hour dietary recall will be collected at the subject’s home within the week before the baseline blood sample collection. Additional 24-hour dietary recalls will be collected twice each at 4 weeks after enrollment, at 16 and 32 weeks gestation (8 recalls total per subject). | | | | | | | | |
| 1. **Data Storage:** Please complete the following questions regarding data storage: | | | | | | | | |
| a. How will the data be collected and recorded? How will the data be coded to protect personal privacy? | | | | | | | | |
| Data will be collected by study staff on case report forms that are unique for each study visit. Data will be entered monthly into a Microsoft Access database created specifically for this project. All subjects will be given a unique study ID number; only the PIs and Study Coordinator will be able to link personal identifying information to their Study ID. All personal data will be destroyed within 10 years of completing the study. | | | | | | | | |
| b. How will the data be stored during the study? | | | | | | | | |
| The Microsoft Access database will be saved daily via the Children’s Hospital & Research Center at Oakland Server. Hard copies of the data will be stored in locked filing cabinets at the National Institute of Nutrition accessible only by the Study Coordinator and PIs. | | | | | | | | |
| c. Who will have access to the data and the data codes? If data with subject identifiers will be released, specify the person(s) and agencies to whom this information will be released? | | | | | | | | |
| Only the PIs and Study Coordinator will have access to the data and the data codes. Data with subject identifiers will not be released. | | | | | | | | |
| d. What will happen to the data when the study is completed? | | | | | | | | |
| All personal data will be destroyed within 10 years of completing the study. | | | | | | | | |
| Risk and Benefit Assessment | | | | | | | | |
| 1. **Potential Risks and Discomforts:** Describe any potential risks or likely adverse effects of the drugs, biologics, devices or procedures subjects may encounter in the study. State the potential risks – physical, psychological, social, legal or other – connected with the proposed procedures and assess their likelihood and seriousness. | | | | | | | | |
| Anthropometry and questionnaires pose no risks to the subjects. After phlebotomy, the participants may receive a bruise and rarely, an infection at the site of the venipuncture. Experienced phlebotomists who are familiar with study subjects will draw all blood samples to minimize these risks. A maximum of 25 mL (about 2 tbsp) per time point of blood will be drawn from each subject. The minimum time interval between the blood draws will be 18 weeks. | | | | | | | | |
| 1. **Safety Precautions for Minimizing Risks:** Describe the procedures for minimizing any potential risks. Where appropriate, discuss provisions for ensuring necessary medical or professional intervention in the event of adverse effects to the subject. | | | | | | | | |
| Procedures for minimizing potential risks to subjects begins at recruitment with excluding women who have any health problems that would counter indicate participation in the study. Blood draws will be done by trained phlebotomists to prevent discomfort and bruising at the blood draw site. Trained phlebotomists will do all of the blood draws to prevent discomfort and bruising. Hemoglobin and hematocrit will be measured at baseline, 16 and 32 weeks gestation to monitor the women’s risk of anemia during pregnancy.    To prevent the risk of loss of privacy every subject will be assigned a unique ID number. Data will be secured both electronically and in locked file cabinets. | | | | | | | | |
| 1. **Benefit Ratio:** What is the risk benefit ratio of this research, compared with available alternatives? Describe the potential benefits the subjects may receive as a result of their participation in the research and what benefits to society may be expected. **For greater than minimal risk research involving children there must be the prospect of direct benefit to the individual subjects.**   Note: The potential benefits of the research must justify the risks to human subjects. The risk benefit ratio of the research must be at least as favorable for the subjects as that presented by standard treatments for their condition. When comparing the risk/benefit ratio of research with that of available alternatives, the alternative of doing nothing should be included in the analysis. | | | | | | | | |
| The potential benefits for the subjects participating in this study include the study incentive, and the possibility of improving, maternal, fetal and infant health and nutrition. Occasionally, some information from the study will be useful for the individual subject, but most often the information will not directly influence care. The results of these studies will be helpful in directing future recommendations for the timing of dietary interventions for pregnancy. | | | | | | | | |
| 1. **Therapeutic Alternatives:** What therapeutic alternative(s) are reasonably available to potential subjects should they choose not to participate in the study? These may be research or non-research based alternatives. | | | | | | | | |
| Should subjects choose not to participate in the study they may acquire foods used in the study supplement by purchasing animal source food at local markets or producing it at home. | | | | | | | | |
| **Financial Considerations** | | | | | | | | |
| 1. **Payment for Participation:** Describe all plans to compensate subjects, including provision of services, and other reimbursements. Describe the conditions that subjects must fulfill to receive full or partial pro-rated payment. | | | | | | | | |
| All subjects who participate in the screening process will be provided with a $1 appreciation gift. All subjects who participate in the longitudinal trial will be reimbursed at a standard rate to offset incurred expenses associated with the study visits ($3/visit for baseline, 16, 24, 32 wks gestation and $3 for the three infant follow-up visits; or total: $21/subject). An end of study thank you gift will be presented to each subject who completes the study. | | | | | | | | |
| 1. **Financial Obligations of Subjects:** Will subjects have to pay for any of the tests or treatments that they receive as part of the research? Please clarify who will pay for the procedures associated with the study as well as procedures that may be part of standard clinical care. Clarify that insurance and other third-party payers may not cover standard procedures if they are associated with a research project. | | | | | | | | |
| Subjects will not be responsible for paying for any tests or treatments. All procedures will be paid through research funds. | | | | | | | | |
| 1. **Emergency Care and Compensation for Research-Related Injury:** If the research presents an unknown or greater than minimal risk illness/injury, the financial liability for the costs of care associated with the potential research related illness/injury must be specified. If no funds are available, please include language to explain this to potential subjects. | | | | | | | | |
| The research does not present an unknown or greater than minimal risk illness/injury | | | | | | | | |
| Informed Consent | | | | | | | | |
| 1. **Capacity to Consent:** Will all subjects have the capacity to give informed consent? If not, describe the likely range of impairment and explain how, and by whom, their capacity to consent will be determined. | | | | | | | | |
| All subjects will have the capacity to provide informed consent in their native language, Vietnamese. | | | | | | | | |
| 1. **Study Personnel Administering the Consent Process:** Please identify by name and training the individual(s) who will be authorized to describe the research to subjects or their representatives, and to invite their participation. To ensure that subjects give complete informed consent and are able to ask and have answered all questions regarding the nature of their participation, the personnel administering the consent must have appropriate training and background. | | | | | | | | |
| Pham Hong Anh and Hoang Thu Nga will be primarily responsible for consenting the subjects, however Andrew Hall and Tu Ngu will all be available to consent as needed and all will complete the CITI certification courses. | | | | | | | | |
| 1. **Process of Consent:** Please discuss how the consent process will be conducted, describing the following elements: 2. The environment and location where the informed consent will be solicited; 3. Opportunities for the potential subjects to discuss their participation with family or others before signing the consent form; 4. How and by whom it will be determined whether the subject or their legally authorized representatives understand the information provided; and 5. **The types of forms used** (e.g., adult consent form, parental permission form, combined form, translations to other languages, etc.) | | | | | | | | |
| Potentially eligible subjects will be identified through the marriage registration process in the Cam Khe District of Phu Tho Province, Vietnam. Local health station staff and Women’s Union personnel will ask the potentially eligible if she is interested in learning more about a research study. If the potential subject is interested, study personnel will meet with her at her home for a screening appointment. During this meeting, the study coordinator will outline the study and complete the screening questionnaire with her. If she is eligible to participate then the study coordinator will review the adult consent form and answer any questions that the participant might have. If the participant would like to enroll at that time she will be able to, she will be given a copy of the consent form and study staff contact information. If the subject is not ready to consent at that meeting then study staff will contact her within 72 hours to see if she has any questions and to schedule a follow-up meeting with her. To make sure that the subject thoroughly understands the study we will ask her to verbally summarize the study back to us. | | | | | | | | |
| 1. **Assent of Minor:**  For subjects age 7 through 17:  N/A (under 7 or adult) | | | | | | | | |
| a) Considering the subject’s potential capacity and medical condition, what is the suggested age of the minors to provide assent? | | | | | |  | | |
| b) Detail below whether the assent should be in writing (a separate assent form signed by the child), and/or obtained orally. | | | | | | | | |
|  | | | | | | | | |
| c) Would it would be appropriate to include on the consent form a signature block for adolescents who are able to understand the adult consent form **(minimal risk studies only).** | | | | | | | Yes  No | |
| d) Detail any justification for requesting that the IRB waive assent.  N/A | | | | | | | | |
|  | | | | | | | | |
| 1. **Information Withheld from Subjects:**  If any information about the research purpose and design of the study will be withheld from subjects, please explain the non-disclosure and describe plans for post-study de-briefing.  N/A | | | | | | | | |
|  | | | | | | | | |
| Data Analysis | | | | | | | | |
| 1. **Statistical Analysis:** Please delineate the data analysis plans for this study. Include planned statistical analyses and explanation of determination of sample size.   Prior to conducting analyses, data will be examined graphically for outliers and normality. Though we expect these features in some of the data, this process will assure that statistical tests assuming normality are only conducted on normally distributed data. If the data are skewed, the data will be log-transformed to achieve normality. Identifying outliers early will also allow us to further explore unusual events within the data set and to examine their influence on the analysis of a particular outcome. The study is a three-arm intervention with randomization of villages and longitudinal measurements of mothers within villages. Hypotheses and analyses will involve comparisons among the three intervention groups and within-groups longitudinally. Study subjects will be randomized upon entry into the study into one of the 3 study group.  *Fetal Growth, Infant Birth Weight, and Prematurity:* Our primary outcome will assess differences in birth weight among groups, a continuous variable. Clustering of infants by village will be included, and mixed-model analysis of covariance (ANCOVA) will be used to determine differences among the three groups. Birth weight will also be analyzed after accounting for cluster covariates including maternal BMI at entry, age, and incidence of infectious diseases. Placental weight (a continuous variable) and fetal growth should both correlate with birth weight and will be analyzed similarly as a continuous variable (see below). Prematurity is our secondary outcome, a dichotomous variable. Thus, we will determine if the incidence of birth weight and pre-term births in the three groups is statistically different using the chi-square test after accounting for cluster covariates. Fetal growth measurements (biparietal diameter, head circumference, abdominal circumference, femur length and calculated fetal weight) are performed at 16 and 24 weeks and at birth. We will use ANCOVA to look at the increase in each of these indicators. We will also evaluate the trajectories of each of these markers over time. This will include random effects at the village level, as well as a best-fit quadratic curve for the trajectories of each of these markers in each fetus, and it will allow the comparison of velocities of each measurement at particular time intervals during the time interval to birth. To evaluate longitudinal changes in qualitative and quantitative measures of fetal growth within and between groups, we will use general estimating equations (GEE) for repeated measurements within subjects. The model will use a logit link function for qualitative measures. For quantitative measures, the loglinear model will be used. Potential modifiers (e.g., maternal nutritional status, infections, stress, etc.) will be incorporated into the model to assess their association with measures of fetal growth.  *Maternal Nutritional Status:* Analysis of biomarkers of maternal nutritional status (iron, zinc, vitamin A, B_12_ and folate status) will be similar to the longitudinal analysis of fetal growth measures as described above. Clustering by village will be accounted for using the cluster random intercept, and the simple analysis of changes in these biomarkers will be assessed by ANCOVA as well as by the above techniques for fetal growth.  *Maternal Infection Morbidity and Immune Responses:* Maternal infection morbidity is measured at regular intervals as an event and duration in days. First, incidence, or the number of new events divided by the period at risk, and prevalence, the total time affected divided by the period at risk, will be compiled for infection morbidity data. Poisson regression will be used for incidence, adjusting for the number of days at risk, and for prevalence, using quasi-likelihood to adjust for lack of independence due to multiple days per event and multiple events per individual. The longitudinal qualitative and quantitative data [cytokine levels (quantitative), Th1/Th2 ratio (qualitative) will be analyzed as for longitudinal data analyses as described above for fetal growth. We will use Pearson's chi-square to compare qualitative measures for the three groups for significance at the 0.05 level. Fisher's exact test will be used for each pairwise comparison. For quantitative measures, we will use one-way analysis of variance to compare the groups. For significance at the 0.05 level, pairwise comparison will be evaluated using Tukey's test. For quantitative measures that are not normally distributed, the Kruskal-Wallis test will be used to compare groups. If significant differences are detected between groups, the Wilcoxon rank sum test will be used to evaluate pairwise comparisons.  *Infant anthropometric measurements:* (Weight, length, head circumference, abdominal circumference) are longitudinal with four time points between birth and 6 months. First, we will use ANCOVA to look at the increase in each of these indicators; an analysis similar to that of birth weight. The second approach involves evaluation of the trajectories of each of these markers over time. This will include random effects at the village level, as well as a best-fit quadratic curve for the trajectories of each of these markers in each infant, and it will allow the comparison of velocities of each measurement at particular time intervals during the first 6-months of life. | | | | | | | | |
| *Sample Size Calculations:* We calculated sample size for our primary outcome, infant birth weight, using the methods of Hayes and Bennett (36). The following assumptions and estimates were used. Since there are no other studies of pre-conception food-based micronutrient interventions in developing countries, birth weight data from a USA WIC supplemental feeding study (6) in which mothers received food vouchers for 5 to 7 months between two pregnancies in comparison to controls who received food vouchers < 2 months between the pregnancies was used to estimate the effect size of preconception to term ASF supplementation compared to controls in this study. The difference in birth weight observed in the WIC study, 130 g, was used. We believe that this is a conservative estimate since the rural Vietnamese women are likely to be more undernourished than the WIC subjects and, therefore, will benefit more from a nutritional intervention, and, the supplement is designed specifically to fulfill current micronutrient deficiencies in the diets of the Vietnamese women. We assumed a standard deviation of birth weight of 385 g based on a survey of 1200 births in the Cam Khe District in 2005 that reported a mean birth weight of 2950 g in a nutrition education intervention study (9). Based on this, our power is 0.80 to detect a 79 g difference in birth weight.  In a study in rural Vietnam conducted by Dr. Dean, the follow up rate after 3 years of infectious disease monitoring was 94% (37). In our pilot feeding trial, we observed a 17% attrition rate over 6 months of daily supplemental ASF, 92% of which occurred in the first 3 months of study. We expect the attrition rate over time in the current study to be lower than that in the pilot. The pilot sample was reproductive-age women not planning to become pregnant. Nearly half of those who dropped out did so to accept employment or go to school outside of the commune; during pregnancy, they do not do either. According to local customs, newly-wed couples are taken care of by the husband’s parents, where it is the young bride’s responsibility to help around the house and prepare meals. Therefore, we assume an individual attrition rate of 10% for the entire period from marriage to 6 months postpartum (an average of about 13 months per participant). Assuming a 95% pregnancy rate within the first year of marriage (based on Cam Khe records for 2008), an average of about 313 mother/infant cohorts per group will be available for analysis at 6 months postpartum.  Based on these assumptions, our study provides 80% power to detect a 79 g difference in birth weight between the PC-T and control groups. The lack of any studies comparing ASF supplementation before versus during pregnancy makes it impossible to estimate the power we will have to detect that difference. If we assume that the difference in birth weight between these two groups is 100 g, based on the study of WIC participants (6), we will have adequate power to detect a difference in birth weight between these two groups in our sample of 1044 women. Our actual power may be higher than observed in the WIC study since the PC-T group will receive the ASF supplement for several months prior to conception as well as during the first months of pregnancy; early pregnancy supplements were not provided to the WIC women. Thus, we expect to find that PC-T supplementation will have a greater effect on birth weight than will MG-T supplementation.  Our secondary outcome is the incidence of prematurity, a dichotomous variable. The rate of preterm deliveries among rural Vietnamese has been reported to be extremely high, about 36% (12). Previous data showing that maternal iron and zinc status affect the rate of preterm deliveries plus the impact of maternal infections on prematurity provide a strong rationale for assessing the effects of ASF supplementation on the prematurity rate in this population (38-40). With the above design and assuming a 35% prematurity rate in the no supplement, control group, we will have 80% power to detect prematurity rates of 23% or less (i.e., a 12% difference or more) between the control and other two ASF intervention groups. If a pair of study groups has prematurity rates ranges between 10% and 30%, we will be able to detect differences of 10% or more with approximately 80% power. | | | | | | | | |

| Comments/Remarks |
| --- |
| This area may be used as continuation of other items. You may also attach additional sheets as necessary. |
|  |

| Attachments |
| --- |
| Please list [Attachments, Supplements and Appendices](http://www.research.ucsf.edu/chr/forms/chrNewAppAtt.asp), including Version(s) and date(s). |
| Screening and Health History Questionnaire  Study Questionnaire  Maternal Anthropometry Form  Supplement Log  Birth Form  Infant Anthropometry Form  Infant Feeding and Morbidity Form  Supplement Composition 10-day Cycle  Consent Form  Table 1: Data Collection Summary by Timeline  Citation List |
|  |

| Study Abstract (For CHORI Website) |
| --- |
| Provide an abstract/full synopsis of this study to be posted on the CHORI Website.  <http://www.chori.org/Human_Research/Human_Research/active_studies_home.html>  It has been known for over 80 years that maternal starvation reduces fetal growth and increases neonatal infections. Consequently, programs have been initiated to improve the fetal nutrient supply by providing food and/or micronutrient supplements to women after they become pregnant. The impact of these efforts has been disappointing. In developing countries, the prevalence of low birth weight (LBW) and infant mortality remain high; ~16% of newborns are LBW and the infant mortality rate is >50/1000 live births. More recently, as a result of improved access to cereals following the ‘green revolution,’ nutritional concerns shifted from protein and energy to micronutrient deficiencies, especially those in animal-source foods (ASFs)—iron, zinc, vitamins A and B_12_. But, infant deaths are still high in rural areas where the women suffer from malnutrition and deliver under-grown infants who are vulnerable to disease. The diets of these women are usually limited to rice and a few vegetables, and they lack key nutrients known to reduce preterm delivery, to support fetal growth, and to prevent infections leading to early deaths. Four of those essential nutrients for good pregnancy outcomes are iron, zinc, vitamin A, folate, and vitamin B_12_.  The well-established Vietnamese VAC system for supporting local production of fish, pork, poultry, and eggs by rural farms provides an opportunity to assess the impact of a food-based, micronutrient-rich supplement on pregnancy outcome in high-risk, rural Vietnamese women. Since maternal nutritional status at conception is strongly linked to pregnancy outcomes, we plan to compare the effect of consuming a micronutrient-rich, animal-source food (ASF) supplement from pre-conception to term with a supplement from mid-gestation to term on infant birth weight, prematurity rate, and infant growth during the first 6 months of life. Maternal nutritional status, anemia, and incidence of infections will also be measured. A total of 1044 women will be recruited from 174 villages at the time of registration for marriage and randomly assigned to one of three groups: I) ASF supplement (~150 kcal, ~14 mg iron, 5.2 mg zinc, 1100 µg RAE vitamin A, and 9 µg vitamin B_12_) 5 days/wk from marriage to term (~13 months); II) ASF supplement 5 days/wk from 16 wks gestation to term (~5 months); or III) routine prenatal care. The ASF supplement, which will be made daily using local foods, will be designed to increase the woman’s intake of iron by 150%, zinc by 70%, vitamins A and B_12_ by 200% and 300%, and folate by 180%, respectively. Maternal dietary intakes, height, weight, mid-upper arm circumference, triceps and subscapular skinfold thickness, iron, zinc, vitamin A and B_12_, and folate status, and immune function will be measured at recruitment, 16, and 34 weeks gestation. Because of the potential impact of infections on fetal growth and maternal health postpartum, the incidence of infections [urinary tract infection (UTI), pneumonia, and diarrhea] will be assessed twice-monthly from enrollment through six months postpartum by a standardized questionnaire administered by a health worker to all three groups in addition to a rapid evaluation of urine for infection. Infant weight, length, head, mid-arm, and abdominal circumference will be measured at birth, 2, 4, and 6 months. The incidence of health problems in the mother and child will be determined from a medical chart review.  This study will be the first to compare a food-based, micronutrient-rich supplement consumed prior to conception to term with one given only during pregnancy. Although it is recognized by many that pregnancy may be too narrow a window to improve maternal nutritional health, it is typical for micronutrient supplements to only be given from the time of enrolling for prenatal care to term. Thus, our results will have world-wide implications as to when maternal supplementation will have the greatest impact on pregnancy outcome in undernourished women. |

**Citation List:**
